# Positive Selection of TLR2 and MyD88 Genes Provides Insights Into the Molecular Basis of Immunological Adaptation in Amphibians

**DOI:** 10.1002/ece3.70723

**Published:** 2024-12-16

**Authors:** Jie Zhang, Ruinan Zhao, Hongyan Bi, Jiaoying He, Yang Guo, Dian Liu, Ganggang Yang, Xiaohong Chen, Zhuo Chen

**Affiliations:** ^1^ The Observation and Research Field Station of Taihang Mountain Forest Ecosystems of Henan Province, College of Life Sciences Henan Normal University Xinxiang China; ^2^ College of Fisheries Henan Normal University Xinxiang China

**Keywords:** amphibian, evolutionary patterns, expression patterns, MyD88, TLR2, vertebrate, *Zhangixalus dennysi*

## Abstract

The transition from water to land of amphibians is evolutionarily significant in the history of vertebrates, and immunological adaptation is an important challenge for amphibians to respond to the dramatic changes of the environmental pathogens during their origin and diversification. Toll‐like receptors (TLRs) are important pattern recognition receptors for the innate immune response and TLRs signaling pathway play essential roles in the immune responses to pathogens and inflammatory reaction. However, the evolutionary patterns and molecular mechanisms underlying their adaptation in amphibians are poorly documented to date. Here, we determined the coding regions, expression patterns of TLR2 and Myeloid differentiation factor 88 (MyD88) in the large treefrog ( *Zhangixalus dennysi* ), and explored the evolutionary patterns of these two genes in amphibians. Quantitative Real‐time PCR analyses showed that the TLR2 and MyD88 mRNA were expressed in all the organs/tissues examined, both with the highest levels in the heart and the lowest levels in the body fat for TLR2 and lung for MyD88. The highly conservation and functional significance of these two genes in amphibians were supported based on the sequence characteristics and evolutionary analyses. Significantly positive selection was found to be acting on TLR2 and MyD88 in amphibians based on different site models. Strong signal of positive selection among different amphibian lineages for these two genes was also detected and a series of positively selected sites were identified based on the branch‐site analysis. Our results suggest that amphibians have adapted to different pathogenic microorganisms during their transition from the aquatic to terrestrial environment and diversification into various habitats. The present study will provide new insights into the evolutionary process and molecular basis underlying the immunological adaptation in vertebrates.

## Graphical abstract

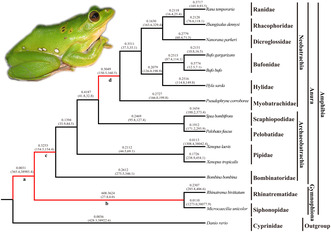

## 1 Introduction

Vertebrates have evolved the innate and adaptive immune systems to eliminate invading pathogenic microorganisms (i.e., bacteria, fungi, protozoa, and viruses) (Akira, Takeda, and Kaisho 2001; Akira, Uematsu, and Takeuchi 2006). Unlike the adaptive immune system only found in vertebrates, the innate immune system (the first line of defense against invasion by pathogens) is the most ancient and universal form of host defense in invertebrates and vertebrates (Akira, Uematsu, and Takeuchi 2006; Rauta et al. 2014). The innate immune responses are triggered upon pathogen recognition by a set of germline‐encoded pattern recognition receptors (PRRs) that recognize the conserved pathogen‐associated molecular patterns (PAMPs) of invading pathogens (Janeway and Medzhitov 2002; Akira, Uematsu, and Takeuchi 2006). As the first and best characterized PRRs, toll‐like receptors (TLRs) can recognize various PAMPs and trigger the signaling pathways that activate immune cells in response to pathogen infection via two primary pathways, that is, the myeloid differentiation factor 88 (MyD88)‐dependent and the MyD88‐independent pathways (Akira, Uematsu, and Takeuchi 2006; Jenkins and Mansell 2010; Takeuchi and Akira 2010; Zhang et al. 2014).

TLRs are type I trans‐membrane glycoproteins that share the same basic architecture comprising of a N‐terminal leucine‐rich repeat (LRR) domain involved in recognition of PAMPs, a trans‐membrane domain responsible for membrane receptor stabilization and receptor‐receptor oligomerization, and a cytoplasmic toll/interleukin‐1 receptor (TIR) domain responsible for signal transduction (Medzhitov, Preston‐Hurlburt, and Janeway Jr. 1997; Takeda, Kaisho, and Akira 2003; Mikami et al. 2012; Gay et al. 2014). Although generally conserved in the structure, the types and numbers of TLRs varied in different vertebrate groups (Voogdt and van Putten 2016), with 13 TLRs in mammals (Xu et al. 2019), 10 in birds (Velová et al. 2018), 10 in reptiles (Priyam et al. 2018), 21 in amphibians (Babik et al. 2014) and 22 in fish (Zhang et al. 2014). Episodes of gene duplication, gene loss and gene conversion might contribute to the different TLR repertoire and functional diversification in different vertebrate species (Hughes and Piontkivska 2008; Roach et al. 2013). For example, the absence of TLR21 and TLR22 in land‐dwelling vertebrates suggested the adaptation of their immune system to different pathogens during the transition from water to land (Oshiumi et al. 2008; Shen et al. 2012; Ishengoma and Agaba 2017). TLRs have long been regarded to be evolutionarily conserved and under strong intense functional constraint (Medzhitov, Preston‐Hurlburt, and Janeway Jr. 1997; Roach et al. 2005; Barreiro et al. 2009; Mukherjee et al. 2009; Mukherjee, Ganguli, and Majumder 2014). Nevertheless, adaptive evolution have been detected in different TLRs in primates (Wlasiuk and Nachman 2010), cetaceans (Shen et al. 2012; Xu et al. 2019), birds (Velová et al. 2018), and reptiles (Priyam et al. 2018). Considering the limited loci examined and the lack of key groups in the evolution of vertebrates, a clear picture of the evolution of the TLR gene family in vertebrates has not been painted so far.

Amphibians, including frogs, toads, salamanders and newts, are a group of evolutionarily significant vertebrates with a history of transition from water to land and subsequent adaptations to a wide variety of habitats (Fei et al. 2009). Immunological adaptation is an important challenge for amphibians to respond to the dramatic changes of the environmental pathogens during their origin and diversification (Fei et al. 2009; Grogan et al. 2018). However, the functions of the adaptive immune system of amphibians are imperfect, and the innate immunity plays important roles in their disease resistance (Grogan et al. 2018). Therefore, amphibians could be one of the most ideal candidate groups for studying the evolutionary process and the associated driving mechanisms of vertebrate innate immune systems. However, the evolutionary patterns of the TLRs and the key genes in the related signal pathway in amphibians are poorly documented to date.

Here, TLR2 and MyD88 were used as an example to reveal the evolutionary history of pattern recognition receptors across amphibians. TLR2 is expressed on the outer membrane and forms heterodimer complex with TLR1 or TLR6 (Takeda, Kaisho, and Akira 2003) to recognize lipoproteins/lipopeptides, peptidoglycans, lipoteichoic acid from gram‐positive bacteria and lipopolysaccharide (LPS) from gram‐negative bacteria (Takeda, Kaisho, and Akira 2003), lipoarabinomannan from myco‐bacteria, zymosan from fungi (Samanta et al. 2012), tGPI‐mucin from *Trypanosoma cruzi* (Debierre‐Grockiego et al. 2007) and hemagglutinin protein from measles virus (Hirschfeld et al. 2001). TLR2 triggers a signaling cascade via the MyD88‐dependent pathway (Zhang et al. 2014). MyD88 is a signal adaptor molecule involved in most TLR pathways, and is a critical target molecule for downstream signal transduction (Medzhitov et al. 1998; Zhang et al. 2019). Given the important roles of the TLR2 and MyD88 genes in the innate immune system, the current study firstly determined the full open reading frame (ORF) sequences of these two genes from representative tree frog, *Zhangixalus dennysi* (Rhacophoridae: *Zhangixalus*), and their expression patterns. We also compared the ORF orthologous sequences of TLR2 and MyD88 within amphibians. The goal of the present study was to test whether evolutionary changes of TLR2 and MyD88 were associated with the transition from water to land and the immunological adaptation of amphibians to various habitats during their diversification. This study will provide new insights into the evolutionary process and molecular basis underlying the immunological adaptation in vertebrates.

## 2 Materials and Methods

### 2.1 Sampling

The large treefrog used in the present study was collected from Jingangtai Mountain, Shangcheng County, Xinyang City, Henan Province (31°41′ N, 115°17′ E). The frogs were euthanized using tricaine methanesulfonate (MS‐222) and sacrificed to collect different organs/tissues. The liver organs/tissues were used for amplify the full TLR2 and MyD88 sequences in *Z. dennysi* , and together with other organs/tissues (i.e., kidney, heart, testicle, pancreas, intestines, stomach, fat body, brain, lung, spleen, skin and muscle) were used for real‐time PCR. All the organs/tissues were dissected out and preserved at −80°C with Sample protector for RNA (TaKaRa, China) for further use. The animal sampling and use protocols of this study were conducted according to all the ethical guidelines and legal requirements in China, and were approved by the Institutional Care and Ethics Committee of Henan Normal University. The study complies with the ARRIVE guidelines for reporting in Vivo Experiments.

### 2.2 RNA Extraction and cDNA Synthesis

Total RNA was isolated from the examined organs/tissues using the RNAiso Plus (TaKaRa, Japan) following the manufacturer's instructions. The RNA integrity and concentrations were determined by electrophoresis on 1% agarose gel and by measuring their absorbance at 260 nm and 280 nm with NanoDrop 2000 spectrophotometer (Thermo Scientific, USA), respectively. The first‐strand cDNAs synthesis were carried out using PrimerScript II 1st Strand cDNA Synthesis Kit (TaKaRa, Japan) for cloning the cDNA sequences and the PrimeScript RT Master Mix (TakaRa, Japan) for quantitative Real‐Time PCR (qRT‐PCR) according to the guidelines of the manufacturer's instructions, respectively. Then the cDNAs were stored at −80°C for further use.

### 2.3 Cloning and Sequencing the Full‐Length cDNA of TLR2 and MyD88

We first designed the degenerate primers of the intermediate fragments of TLR2 and MyD88 via Primer Premier 5.0 (Premier Biosoft International, CA, USA) according to the conserved sequences of TLR2 and MyD88 in other frogs such as *Xenopus tropicalis* , *Nanorana parkeri* , *Rana japonica, R. temporaria* and *R. dybowskii* , and then designed the 5′/3′ RACE primers based on the intermediate fragments of TLR2 and MyD88 amplified. The degenerate primers, gene‐specific primers and adapter primers information were shown in Table S1. Partial sequence amplification, 5′/3′ RACE, purification, cloning and sequencing of the PCR products were performed according to the methods described in Zhu et al. (2001) and Zhang et al. (2017). The cycling protocol was one cycle of 95°C for 5 min, 35 cycles of 95°C for 30 s, 55°C for 30 s, 72°C for 40 s, followed by one cycle of 72°C for 10 min. The 5′ and 3′ RACE were then performed using a SMARTer RACE cDNA amplification kit and SMARTer RACE kit (TaKaRa, China) according to the manufacturer's instructions. The amplified PCR products were purified with a MiniBEST Agarose Gel DNA Extraction Kit (TaKaRa, China), cloned into Pmd18‐T vectors, and then sequenced in both directions using an ABI 3730 automated genetic analyzer (Applied Biosystems) by Shanghai Sangon Biological Engineering Techonology and Service Co. Ltd. Five to six repeated amplifications were conducted and sequenced to confirm its sequence. The same PCR primers were used for sequencing. The newly TLR2 and MyD88 sequences were deposited in GenBank under accession number: PQ118635 and PQ118636.

### 2.4 Sequence Analysis and Protein Structure Prediction

We used Basic Local Alignment Search Tool (BLAST) (http://www.ncbi.nlm.nih.gov/blast/Blast.cgi) to search homologous sequences and confirm the sequences obtained. The program DNASTAR EditSeq (DNASTAR Inc., Madison, WI, USA) was used to determine the ORFs of verified TLR2 and MyD88 genes. EXPASY (http://web.expasy.org/translate/) was used to translate the deduced amino acid sequences. Compute pI/Mw (http://web.expasy.org/compute_pi/) was used to calculate the putative theoretical isoelectric point (pI) and molecular weight (Mw) of the deduced amino acid. The protein domains were predicted using SMART program (http://smart.embl‐heidelberg.de). The three‐dimensional domain structures of TLR2 and MyD88 proteins were predicted using the homology modeling by the Swiss‐Model Server (http://swissmodel.expasy.org/) and analyzed by the software SPDBV 4.01 and POV‐Ray v3.6. The N‐glycosylation site and the phosphorylation site were predicted by the tool NetNGlyc 1.0 (http://www.cbs.dtu.dk/services/NetNGlyc/) and NetPhos 2.0 (http://www.cbs.dtu.dk/services/NetPhos/) Server, respectively. SignalP‐5.0 (h ttp://www.cbs.dtu.dk/services/SignalP/index.php) was used to predict the cleavage site of the signal peptide.

### 2.5 Quantitative Real‐Time PCR (qRT‐PCR)

We used the samples collected in section 2.1 to investigate the expression patterns of TLR2 and MyD88 in 13 different organs/tissues of the large treefrog. The cDNAs synthesized in section 2.2 were fivefold serially diluted using the EASY Dilution (for Real‐Time PCR) (TaKaRa, Japan) and then used as the templates for qRT‐PCR. The primers were designed based on the cDNA sequences obtained in the present study and are shown in Table S1. Each sample was detected in triplicate, and β‐actin was used as a reference gene. The qRT‐PCR was conducted in a reaction mixture of 10 μL consisted of 1 μL of cDNA templates, 0.4 μL of each primer (10 μM), 5 μL of 2 × TB Green Premix EX Taq II (TaKaRa, China), and 3.2 μL of ddH_2_O following the manufacture's protocol with the LightCycler96 Real Time PCR system (Roche, Switzerland). The reaction was performed in condition as follows: 95°C for 10 min, 40 cycles of 95°C for 15 s and 60°C for 1 min, followed by melting curve analysis (95°C for 15 s, 60°C for 1 min, 95°C for 15 s, and 60°C for 15 s) The relative gene expression levels of TLR2 and MyD88 were normalized with the reference gene and analyzed using the 2^−△△Ct^ method and presented as fold changes for the calibrator (Livak and Schmittgen 2001), and then the significances for qRT‐PCR data were assessed using the Unpaired Student's *t*‐test.

### 2.6 Sequence Alignment and Phylogenetic Reconstruction

TLR2 proteins from 41 species (three fishes, 19 amphibians, five reptiles, six birds, and eight mammals) and MyD88 from 34 species (three fishes, 16 amphibians, four reptiles, four birds, and seven mammals) were searched and downloaded from NCBI (http://www.ncbi.nlm.nih.gov/) and were used for phylogenetic reconstruction. Detailed taxonomic and sequence information is present in Table 1. The sequences were aligned using the MACSE v2.06 (Ranwez et al. 2018) implemented in PhyloSuite v1.2.3 (Xiang et al. 2023; Zhang et al. 2020) and the nucleotide sequences alignment was generated based on the putative amino acid sequences alignment. Phylogenetic relationships were determined using the Bayesian inference (BI) and Maximum Likelihood (ML) algorithms in PhyloSuite v1.2.3 (Xiang et al. 2023; Zhang et al. 2020) based on nucleotide datasets. *Carassius auratus* was utilized as outgroup for the phylogenetic analyses according to Wang et al. (2012). The optimal substitution models were selected using ModelFinder v2.2.0 (Kalyaanamoorthy et al. 2017) according to the Akaike Information Criterion (AIC). The best‐fit substitution models retrieved for the BI and the ML analyses of the nucleotide datasets were GTR + I + G + F and TPM3u + R4 + F models for the TLR2 gene, and GTR + I + G + F model and TIM3 + R4 + F models for the MyD88 gene, respectively. Maximum likelihood analyses were conducted using IQ‐TREE (Nguyen et al. 2015) with 5000 ultrafast (Minh, Nguyen, and von Haeseler 2013) bootstraps. Bayesian Inference phylogenies were inferred using MrBayes v3.2.7a (Ronquist et al. 2012) and twenty million generations were run with four Markov chains. The first 25% of the trees were deleted as burn‐in and the remaining trees were used to generate the consensus tree and calculate Bayesian posterior probabilities (PP). Tracer v1.4 (Rambaut and Drummond 2007) was used to determine the stationarity of the likelihood scores of sampled trees.

**TABLE 1:** List of taxonomic samples and sequences used in this study.

Class	Order	Family	Scientific name	Accession number	
				TLR2	MyD88
Mammalia	Rodentia	Sciuridae	*Marmota monax*	XM_046458955.2	XM_046436604.2
		Circetidae	*Phodopus roborovskii*	XM_051199017.1	XM_051202282.1
		Muridae	*Mus musculus*	NM_011905.3	NM_010851.3
	Artiodactyla	Bovidae	*Ovis aries*	DQ890157.1	NM_001166183.1
		Bovidae	*Bubalus bubalis*	KU984439.1	JN608794.1
	Primates	Hominidae	*Homo sapiens*	NM_001318796.2	U84408.1
	Carnivora	Mustelidae	*Gulo gulo luscus*	OM291788.1	—
	Scandentia	Tupaiidae	*Tupaia chinensis*	KT354317.1	XM_027768468.1
Aves	Passeriformes	Corvidae	*Corvus cornix cornix*	XM_019293461.2	XM_010394992.4
		Pipridae	*Pipra filicauda*	XM_039384651.1	XM_027733453.2
	Anseriformes	Anatidae	*Anas platyrhynchos*	KX687002.1	NM_001310832.1
	Galliformes	Phasianidae	*Gallus gallus*	NM_001161650.3	NM_001030962.5
	Columbiformes	Columbidae	*Columba livia*	XM_013372151.3	—
	Cuculiformes	Cuculidae	*Cuculus canorus*	XM_054065790.1	—
Reptilia	Squamata	Colubridae	*Pantherophis guttatus*	XM_034411407.2	XM_034401653.2
			*Ahaetulla prasina*	XM_058193135.1	XM_058182315.1
		Dactyloidae	*Anolis carolinensis*	XM_062981094.1	XM_003221868.4
		Viperidae	*Protobothrops mucrosquamatus*	XM_015818356.1	XM_015832556.1
	Crocodylinae	Alligatoridae	*Alligator sinensis*	XM_006019140.1	—
Amphibia	Anura	Ranidae	***Rana temporaria***	XM_040331977.1	XM_040352785.1
			***Rana dybowskii***	—	KU363754.1
			*Rana tagoi tagoi*	MG999550.1	—
			*Rana ornativentris*	MG999542.1	—
			*Rana japonica*	MG999532.1	—
			*Odorrana amamiensis*	MH165314.1	—
			*Odorrana ishikawae*	MH165315	—
			***Aquarana catesbeiana***	—	OL589125.1
		Rhacophoridae	***Zhangixalus dennysi***	PQ118635	PQ118636
		Dicroglossidae	***Nanorana parkeri***	XM_018557931.1	XM_018553493.1
		Bufonidae	***Bufo gargarizans***	XM_044300268.1	XM_044293665.1
			***Bufo bufo***	XM_040418560.1	XM_040433776.1
		Hylidae	***Hyla sarda***	XM_056562456.1	XM_056519669.1
		Scaphiopodidae	***Spea bombifrons***	XM_053455377.1	XM_053466761.1
		Myobatrachidae	***Pseudophryne corroboree***	XM_063920519.1	XM_063921817.1
		Pelobatidae	***Pelobates fuscus***	XM_063458536.1	XM_063450823.1
		Pipidae	***Xenopus laevis***	XM_018233556.2	AF294272.1
			***Xenopus tropicalis***	XM_004911150.4	NM_001016837.2
		Bombinatoridae	***Bombina bombina***	XM_053703754.1	XM_053713779.1
	Gymnophiona	Rhinatrematidae	***Rhinatrema bivittatum***	XM_029610508.1	XM_029588377.1
		Siphonopidae	***Microcaecilia unicolor***	XM_030188155.1	XM_030197984.1
	Caudata	Cryptobranchidae	***Andrias davidianus***	—	KC152963.1
Pisces	Cypriniformes	Cyprinidae	*Carassius auratus*	MK292330.1	MK246404.1
			***Danio rerio***	NM_212812.1	NM_212814.2
			*Megalobrama amblycephala*	XM_048197780.1	XM_048174478.1

*Note:* The species names in bold letter were used for the analysis of selective pressure.

### 2.7 Analysis of Selective Pressure

The useful measurement for identifying adaptive evolution is to estimate the nonsynonymous (*d*_*N*_)/synonymous substitution (*d*_*S*_) rate (*ω* = *d*_*N*_/*d*_*S*_), where *ω* = 1, *ω* < 1, and *ω* > 1 correspond to neutral evolution, purifying and positive selection, respectively (Yang 2007; Xu et al. 2019). To determine the evolutionary patterns of the TLR2 and MyD88 in amphibians during their transition from water to land and their diversification, we estimated the *ω* ratio using two maximum likelihood frameworks, the CODEML program in PAML 4.7 (Yang 2007) and HyPhy package via the Datamonkey webserver (https://www.datamonkey.org/) (Kosakovsky Pond and Frost 2005; Murrell et al. 2012). Only the amphibian representatives with the full‐length form for TLR2 (fourteen amphibians) and MyD88 (seventeen amphibians) were used for the analysis of selective pressure (Table 1). The well‐supported phylogeny of amphibians (Portik, Streicher, and Wiens 2023) was used as the guide tree for the subsequent analyses.

To identify the probabilities of sites under positive selection in each gene for amphibian species being examined, three pairs of site models implemented in the CODEML were tested: M1a (nearly neutral: *ω*_*0*_ < 1 and *ω*_*1*_ = 1) versus M2a (positive selection: *ω*_*0*_ < 1, *ω*_*1*_ = 1 and *ω*_*2*_ > 1), M7 (beta distribution: 0 < *ω*_*0*_ < 1) and M8 (positive selection; beta distribution: 0 < *ω*_*0*_ < 1 and *ω*_*1*_ > 1), and M8a (nearly neutral; beta distribution: 0 < *ω*_*0*_ < 1 and *ω*_*1*_ = 1) versus M8 (Yang et al. 2000; Yang, Wong, and Nielsen 2005). All the positively selected sites in site models were determined via Bayes Empirical Bayes analysis with posterior probabilities ≥ 0.80 (Yang 2007). In addition, positively selected sites were also identified by the fixed‐effect likelihood (FEL) model, mixed effects model of evolution (MEME) and single‐likelihood ancestor counting (SLAC) implemented in HyPhy package (Kosakovsky Pond and Frost 2005; Murrell et al. 2012) using the Datamonkey webserver (https://www.datamonkey.org/). The FEL model assumes the *d*_*N*_/*d*_*S*_ values on a site‐by‐site basis, without assuming a priori distribution of rates across sites. The MEME model identifies instances of both episodic and pervasive positive selection at the level of an individual site. The SLAC model calculates the expected and observed numbers of synonymous and nonsynonymous substitutions to infer selection. Sites with *p* values < 0.2 for FEL, < 0.1 for SLAC and MEME models were considered as candidates under positive selection. Only codons identified by at least two of the above methods used were considered to be under positive selection (Xu et al. 2019). Amino acid substitutions can be either conservative or radical according to their change in a certain physicochemical property (Xu et al. 2019). The amino acid properties conserved or changed that occurred in the positively selected sites were inferred using TreeSAAP (Woolley et al. 2003).

To determine whether or not the focal branch had undergone positive selection, we also used the branch, branch‐site and clade models (Yang and Nielsen 2002). For branch‐specific models, the ‘free‐ratio’ (M1) model (which assumes an independent ω ratio for each branch) versus the ‘one‐ratio’ (M0) model (which assumes the same ω for all branches) (Yang 1998), and the two‐ratio model (which allows ω to differ between the background and a focal branch) versus M0 model were conducted (Yang and Nielsen 2002). Modified branch‐site model A (test 2) (model = 2, Nsites = 2) was performed for each gene in each foreground lineage (details on test 2 in Zhang, Nielsen, and Yang 2005). The null model for test 2 is the branch‐site model A with ω_2_ = 1 fixed. The clade model C versus M1a was also used to detect divergent selection acting on groups of related key taxa. The clade Model C includes two clades (i.e., focal clade and background clade) and three site classes (0, 1, and 2). Purifying selection (0 < *ω*_*0*_ < 1) and neutral selection (*ω*_*1*_ = 1) were assumed in site class 0 and 1, whereas branches in the two clades are evolving with ω_2_ and ω_3_ (*ω*_*2* ≠_ *ω*_*3*_) in site class 2, respectively. Clade models were separately performed for the ancestral Amphibia, Caudata, Gymnophiona, Anura and Neobatrachia. The likelihood ratio tests (LRTs) statistic (2ΔL) approximates to a chi‐square distribution and was used to compare nested likelihood models with the number of degrees of freedom equal to the difference in the numbers of free parameters between models.

## 3 Results

### 3.1 Cloning and Characterization of the cDNA Sequences of ZdTLR2 and ZdMyD88

**FIGURE 1:**
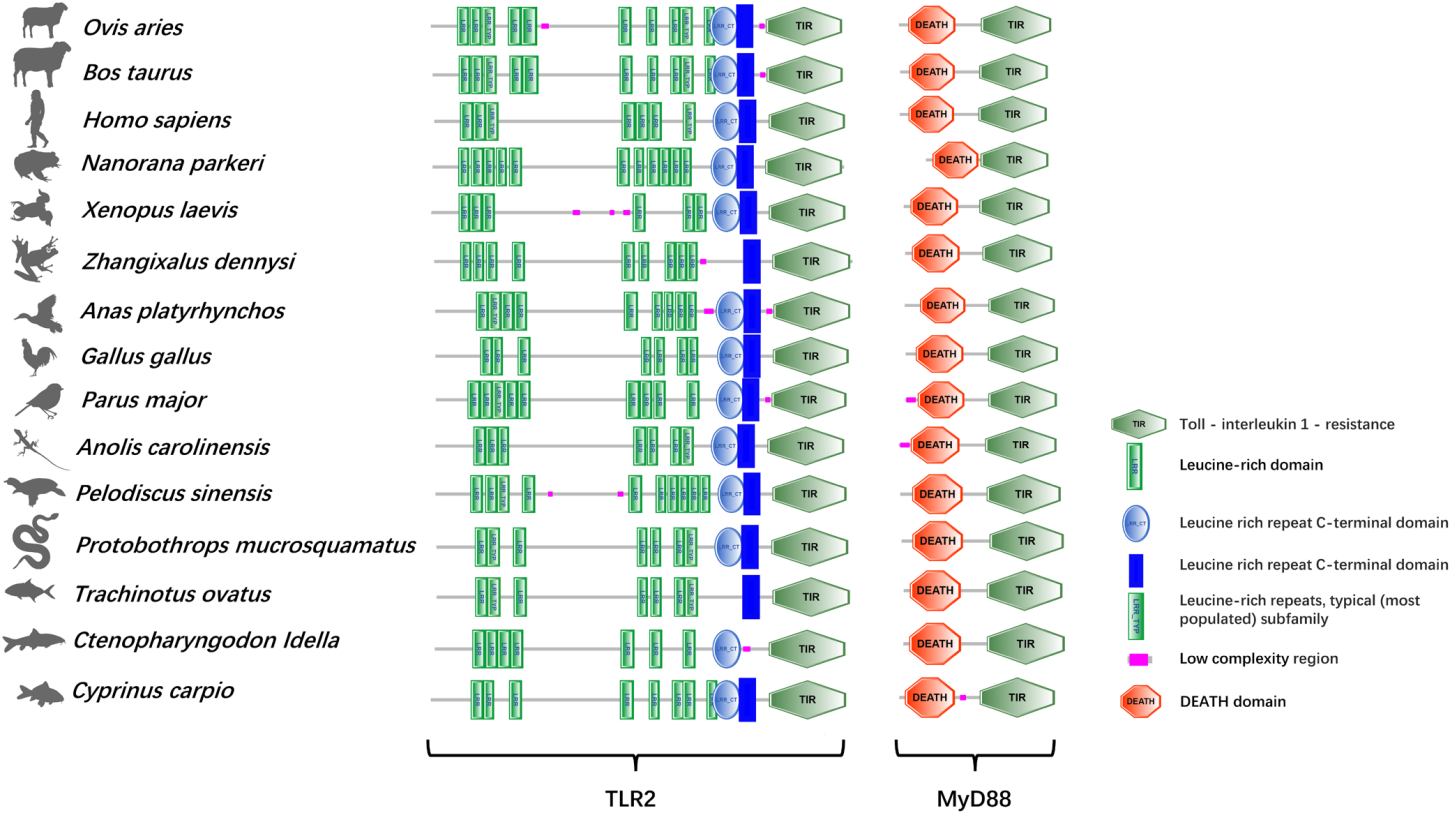
Comparison of the TLR2 and MyD88 domain structures among representative vertebrates. The domain organization was predicted using the SMART programs, and the sequences used for comparison are listed in Table 1. The species silhouette are retrieved from PhyloPic (https://beta.phylopic.org/). LRR: Leucine‐rich repeat; TIR: Toll/IL‐1 Receptor.

**FIGURE 2:**
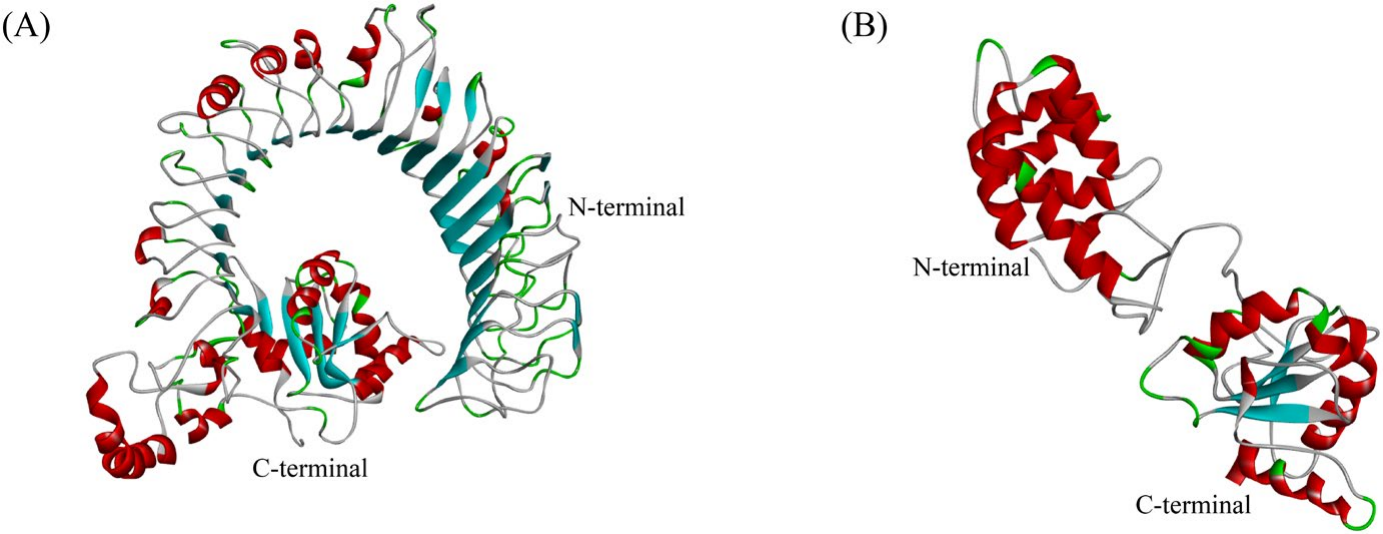
Three‐dimensional structure of the ZdTLR2 (A) and ZdMyD88 (B).

The full‐length cDNA of TLR2 and MyD88 were cloned and characterized in the large treefrog ( *Z. dennysi* ), termed as ZdTLR2 and ZdMyD88. The full‐length cDNA of *ZdTLR2* was 2546 bp, including a 76 bp 5′‐untranslated region (UTR), a 15 bp 3′‐UTR, and a 2361 bp ORF region (Figure S1). The ZdTLR2 ORF encodes a predicted protein of 786 amino acids, with an estimated molecular weight of 89.9 kDa and a theoretical isoelectric point of 5.83 (Figure S1). The putative ZdTLR2 protein contained 11 N‐glycosylation sites and 53 phosphorylation sites, including 32 serine, 17 threonine and 4 tyrosine residues (Figure S1). SMART predicated that ZdTLR2 possess a typical TLR domain architecture with a signal peptide (residues 1–25), nine LRRs (residues 50–494), a trans‐membrane region (residues 586–608) and a C‐terminal TIR domain (residues 636–781) (Figures 1 and S1). As shown in Figure 1, the ZdTLR2 domain structure is similar to that of other vertebrate TLR2, although the number of LRR domains varied in different vertebrates. The secondary structures of ZdTLR2 protein is mainly α‐helix (accounting for 49.49%), whereas the proportion of β‐sheet is low (3.31%) (Figure S1). The tertiary structure of ZdTLR2 is shown in Figure 2A and the extracellular domain presents the characteristic horseshoe shape.

Regarding ZdMyD88, the full‐length cDNA was identified to have 1472 bp, consisting of a 54 bp 5′‐UTR, a 210 bp 3′‐UTR and a 861 bp ORF encoding a protein of 286 amino acids with a calculated molecular mass of 32.8 kDa and a theoretical isoelectric point of 5.83 (Figure S2). SMART analysis showed that ZdMyD88 contained the characteristic N‐terminal death domain (residues 14–105) and C‐terminal TIR domain (residues 151–285) as found in other vertebrates (Figures 1 and S2); however, no corresponding signal peptide sites were detected for ZdMyD88 (Figures 2B and S2). The putative ZdMyD88 protein contained one N‐ glycosylation site and 28 phosphorylation sites, including 14 serine, 11 threonine and 3 tyrosine residues (Figure S2). Sopma was used to predict the secondary structure of ZdMyD88 protein, and it was found that the α‐helix accounted for 43.36% and the β‐sheet accounted for 3.85%, showing random curl (Figure S2). The 3D model of ZdMyD88 consisted of 19 β‐sheets and 13 α‐helices (Figure 2B).

### 3.2 ZdTLR2 And ZdMyD88 Gene Expression Patterns in Different Tissues

**FIGURE 3:**
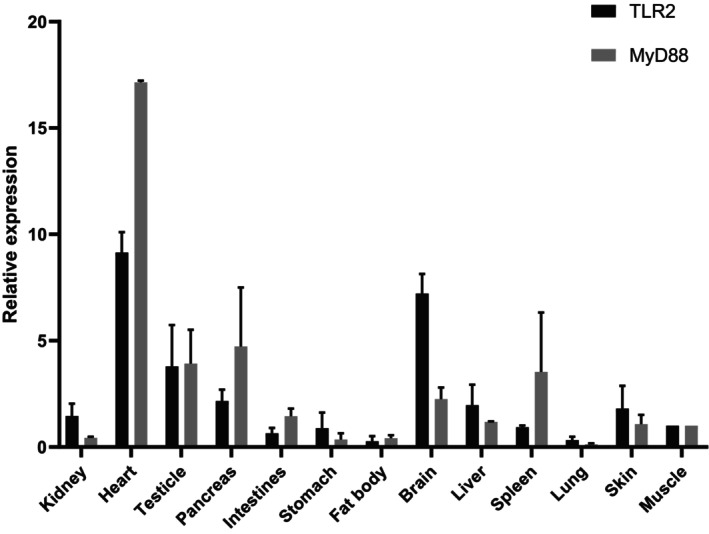
Expression analysis of ZdTLR2 (black color) and ZdMyD88 (gray color) in different tissues. The mRNA expression levels of target genes relative to β‐Actin were analyzed using the 2^−ΔΔCt^ method and gene expression in fertilized eggs was chosen as calibrator (set as 1). The relative expression of ZdTLR2 and ZdMyD88 were presented as fold change for the calibrator. All data were expressed as mean ± standard deviation (*n* = 3).

Quantitative RT‐PCR was performed to detect the mRNA expression levels of ZdTLR2 and ZdMyD88 genes in thirteen organs/tissues (i.e., kidney, heart, testicle, pancreas, intestines, stomach, fat body, brain, lung, spleen, liver, skin and muscle). As shown in Figure 3, ZdTLR2 and ZdMyD88 showed a broad expression distribution with i expression patterns varying greatly among the different organs/tissues. ZdTLR2 presents higher expression in the heart, followed by the brain, testis, and pancreas, and the lowest expression was observed in fat body (Figure 3). The highest expression of ZdMyD88 was also detected in the heart, followed by the pancreas, testis and spleen, and the lowest expression was in lung tissue (Figure 3).

### 3.3 Alignments and Phylogenetic Analysis of TLR2 and MyD88

A total of 41 TLR2 genes and 34 MyD88 genes were obtained and aligned for phylogenetic reconstruction. For TLR2, the same basic architecture comprising of an N‐terminal leucine‐rich repeat (LRR) domain, a trans‐membrane domain, and a cytoplasmic toll/interleukin‐1 receptor (TIR) domain was observed for all the vertebrates examined despite the predicted numbers of LRRs varied among these species (Figure 1). For MyD88, similar N‐terminal death domain and C‐terminal TIR domain were predicted for all the species examined (Figure 1). The evolutionary history of vertebrate TLR2 and MyD88 were reconstructed using ML and BI analysis of nucleotide datasets, which produced similar topologies for both genes with good support (Figures S3 and S4). The monophyly of the tetrapods, reptiles, birds and mammals were strongly supported (PP = 1.0, BP = 100) for both genes, whereas the monophyly of amphibians was revealed only for MyD88 gene (Figure S4). Representatives of birds and reptiles were grouped as the sister taxa to mammals with strong supports based on the analyses of both genes (Figures S3 and S4). Phylogenetic relationships of the amphibian families examined were concordant with those in previous studies (Feng et al. 2017; Portik, Streicher, and Wiens 2023) except the basal position of *X. laevis* for TLR2 (Figure S3) and the sister relationship of Myobatrachidae to Ranidae and Rhacophoridae for MyD88 (Figure S4). Members of the sampled neobatrachian families (i.e., Ranidae, Rhacophoridae, Dicroglossidae, Bufonidae, Hylidae, Myobatrachidae) were found to be monophyletic with strong support for both genes (Figures S3 and S4). Representatives of Ranidae and Rhacophoridae were grouped with strong support and they appeared as the sister taxa to Dicroglossidae for TLR2 (Figure S3).

### 3.4 Positive Selection for Amphibian TLR2 and MyD88

**FIGURE 4:**
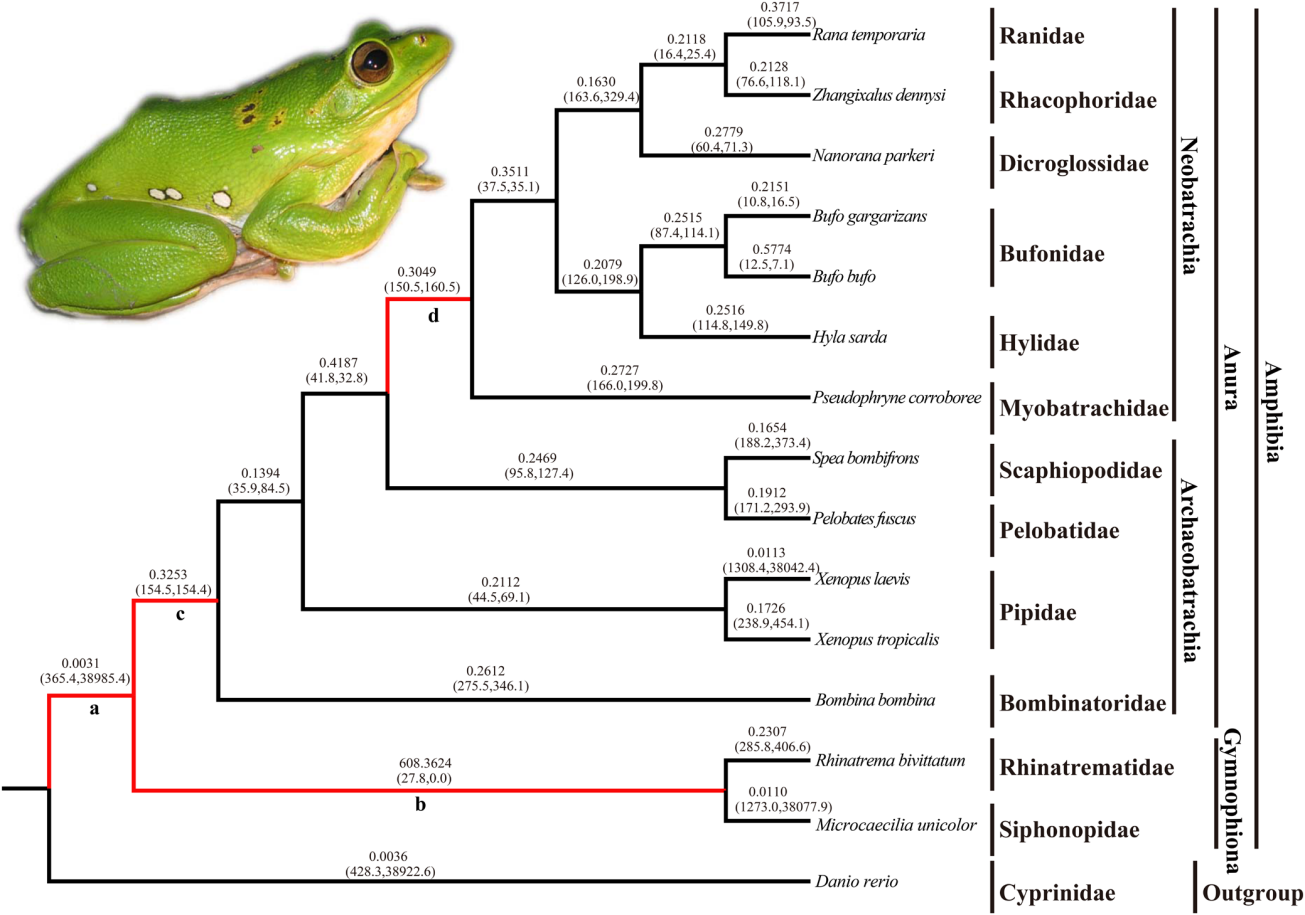
The *ω* values of TLR2 genes in distinct evolutionary lineages of amphibians. Branches a‐d relate to those shown in Table 2. The ω values of individual branches shown are based on the free‐ratio model. The estimated numbers of nonsynonymous and synonymous changes are shown in parentheses. Photo of *Z. dennysi* is shown.

**FIGURE 5:**
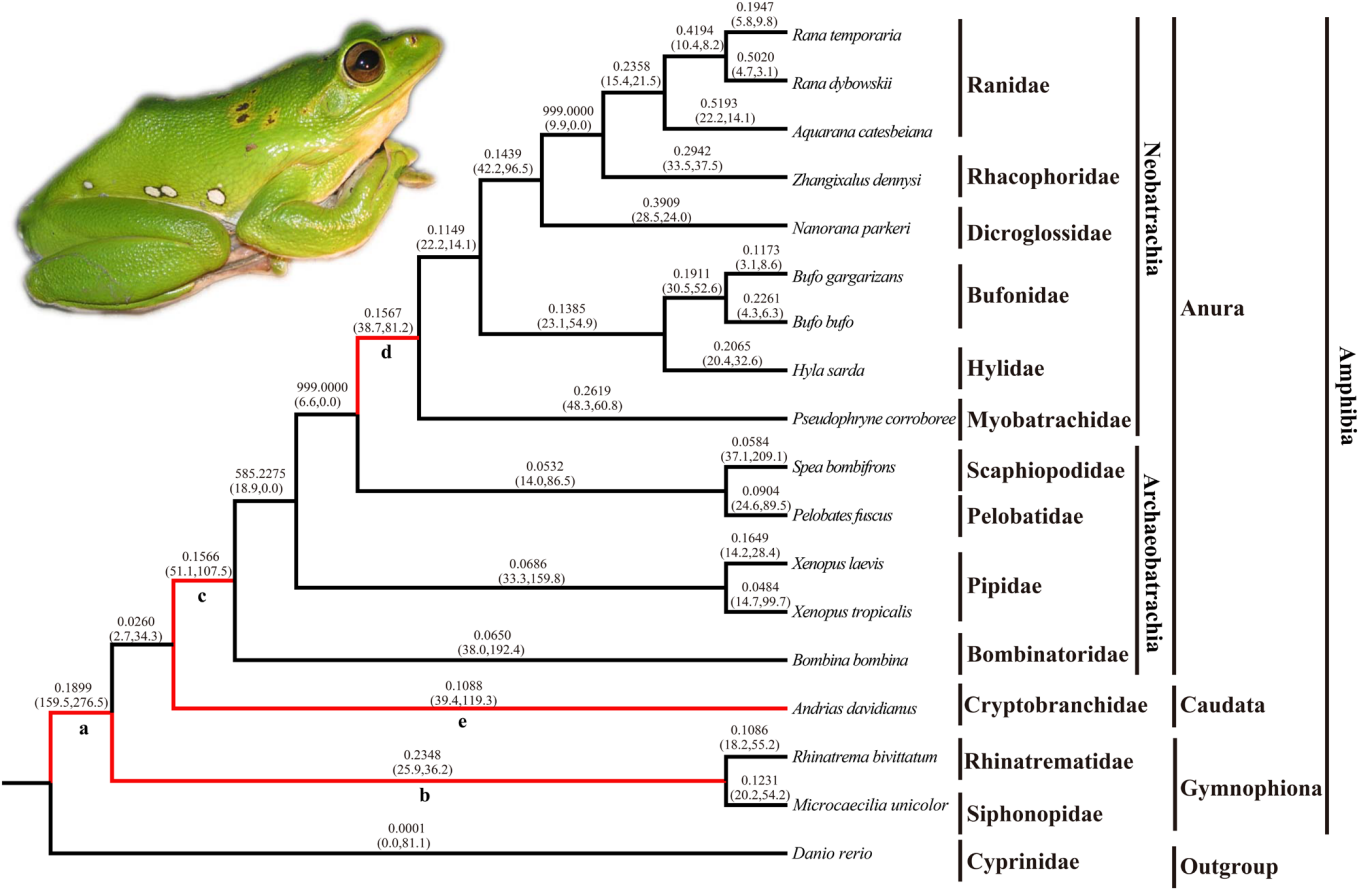
The *ω* values of MyD88 genes in distinct evolutionary lineages of amphibians. Branches a‐e relate to those shown in Table 3. The ω values of individual branches shown are based on the free‐ratio model. The estimated numbers of nonsynonymous and synonymous changes are shown in parentheses. Photo of *Z. dennysi* is shown.

The selective constraints on amphibian TLR2 and MyD88 were analyzed using a series of evolutionary models with the species tree shown in Figures 4 and 5 as the working topology, respectively. In the branch‐specific model analyses, the *ω* ratio calculated in the one‐ratio model (M0) for TLR2 and MyD88 was 0.215 and 0.138 (Tables 2 and 3), respectively, which indicated a generally strong purifying selection for both amphibian TLR2 and MyD88 genes. The free‐ratio model (M1) fitted the data better than did the one‐ratio model for both genes with *p* value of zero for both TLR2 and MyD88, which suggested the different ω ratios among branches for both genes (Tables 2 and 3, Figures 4 and 5). The site model analyses showed that selection model (M8) fit the data significantly better than did neutral model M7 for both genes (*p* = 0.03 for TLR2; *p* = 0.044 for MyD88), whereas model M2a did not fit better than M1a (*p* = 1) (Tables 2 and 3). Five sites (99A, 232 T, 261 N, 287E and 773 T) for TLR2 and two sites (5 L and 141 K) for MyD88 were identified to be under positive selection by the BEB approach with posterior probabilities ≥ 0.80 based on the M8 model (Tables 2 and 3). In addition, the FEL, MEME and SLAC models identified 16, 28 and 315 codons for TLR2 to be under positive selection, respectively (Tables S2, S3 and S4). One and eleven positively selected codons were observed for MyD88 based on the FEL and MEME models, respectively (Tables S5 and S6). When we combined all the methods used, a total of twenty‐four codons for TLR2 and two codons for MyD88 were commonly yielded by at least two methods (Table 4) and these sites were considered as robust candidates of positive selection. It is important to note that all of the 24 positively selected sites for TLR2 and the two sites under positive selection for MyD88 were radical amino acid changes (Table 4).

**TABLE 2:** CODEML analyses of selective pattern on the TLR2 gene in amphibians.

Models	‐lnL	Models compared	2⊿lnL^a^	d.f.^b^	*p* value^c^	Estimate of parameters	Positively selected sites (*p* ^d^)
***Branch models***							
M0:one‐ratio	28218.238					*ω* = 0.215	Not allowed
M1: free‐ratio	28170.128	Mo versus M1	96.220	27	**0.000**	*ω* variation for each branch	Not allowed
***Site models***							
M1a (nearly neutral)	27679.820					*p*_*0*_ = 0.599, *p*_*1*_ = 0.401, *k*(kappa) = 2.040, *ω*_*0*_ = 0.115, *ω*_*1*_ = 1	Not allowed
M2a (positive selection)	27679.820	M2a versus M1a	0	2	1	*p*_*0*_ = 0.599, *p*_*1*_ = 0.158, *p*_*2*_ = 0.243, *k* = 2.040, *ω*_*0*_ = 0.115, *ω*_*1*_ = 1, *ω*_*2*_ = 1	92A (0.850), 232 T (0.934), 261 N (0.874)
M7 (beta)	27315.337					*p* = 0.580, *q* = 1.633, *k* = 1.785	Not allowed
M8 (beta and omega)	27309.346	M8 versus M7	11.982	2	**0.003**	*p*_*0*_ = 0.994, *p*_*1*_ = 0.006, *p* = 0.595, *q* = 1.725, *k* = 1.799, *ω* = **5.667**	99A (0.880), 232 T (0.954), 261 N (0.920), 287E (0.866), 773 T(0.806)
M8a (beta)	27314.696	M8a versus M8	10.7	2	**0.005**	*p*_*0*_ = 0.974, *p*_*1*_ = 0.026, *p* = 0.602, *q* = 1.874, *k* = 1.789, *ω* = 1	
***Branch‐site model***							
**Ancestral Amphibia (branch a)**							
Alternative	27667.716	Alternative versus Null	4.95	1	**0.03**	*ω*_*0*_ = 0.112, *ω*_*1*_ = 1, *ω*_*2*_ = **44.720**	302S (0.824), 337C (0.815), 347C (0.890), 668 K (0.865)
Null	27670.191					*ω*_*0*_ = 0.110, *ω*_*1*_ = 1, *ω*_*2*_ = 1	
**Gymnophiona (branch b)**							
Alternative	27678.866	Alternative versus Null	0.434	1	0.510	*ω*_*0*_ = 0.114, *ω*_*1*_ = 1, *ω*_*2*_ = 1	
Null	27679.083					*ω*_*0*_ = 0.114, *ω*_*1*_ = 1, *ω*_*2*_ = 1	
**Anura (branch c)**							
Alternative	27674.640	Alternative versus Null	10.358	1	**0.001**	*ω*_*0*_ = 0.117, *ω*_*1*_ = 1, *ω*_*2*_ = **463.766**	326Y (0.813)
Null	27679.819					*ω*_*0*_ = 0.115, *ω*_*1*_ = 1, *ω*_*2*_ = 1	
**Neobatrachia (branch d)**							
Alternative	27677.385	Alternative versus Null	3.75	1	**0.005**	*ω*_*0*_ = 0.115, *ω*_*1*_ = 1, *ω*_*2*_ = **9.930**	469E (0.829), 740S (0.920)
Null	27679.260					*ω*_*0*_ = 0.115, *ω*_*1*_ = 1, *ω*_*2*_ = 1	
***Clade model C***							
Ancestral Amphibia (branch a)	27363.939	M1a versus branch c	631.762	3	**0.000**	*ω*_*0*_ = 0.021, *p*_*0*_ = 0.283, *ω*_*1*_ = 1, *p*_*1*_ = 0.168, *ω*_*2*_ = 0.065, *ω*_*3*_ = 0.255, *p*_*2*_ = 0.550	
Gymnophiona (branch b)	27371.285	M1a versus branch e	617.070	3	**0.000**	*ω*_*0*_ = 0.024, *p*_*0*_ = 0.293, *ω*_*1*_ = 1, *p*_*1*_ = 0.159, *ω*_*2*_ = 0.258, *ω*_*3*_ = 0.231, *p*_*2*_ = 0.548	
Anura (branch c)	27367.230	M1a versus branch l	625.18	3	**0.000**	*ω*_*0*_ = 0.022, *p*_*0*_ = 0.291, *ω*_*1*_ = 1, *p*_*1*_ = 0.155, *ω*_*2*_ = 0.183, *ω*_*3*_ = 0.268, *p*_*2*_ = 0.554	
Neobatrachia (branch d)	27370.741	M1a versus branch l	618.158	3	**0.000**	*ω*_*0*_ = 0.024, *p*_*0*_ = 0.294, *ω*_*1*_ = 1, *p*_*1*_ = 0.157, *ω*_*2*_ = 0.243, *ω*_*3*_ = 0.273, *p*_*2*_ = 0.549	

^a^ The log‐likelihood difference between models compared.

^b^ The number of degrees of freedom equal to the difference in the numbers of free parameters between models.

^c^ Likelihood ratio test *p* values were adjusted for multiple testing with a Benjamini and Hochberg's procedure and threshold of 0.05.

^d^ Posterior probabilities of the BEB analysis with *p* > 0.8 considered as candidates of selection.

**TABLE 3:** CODEML analyses of selective pattern on the MyD88 gene in amphibians.

Models	‐lnL	Models compared	2⊿lnL^a^	d.f.^b^	*p* value^c^	Estimate of parameters	Positively selected sites (*p* ^d^)
***Branch models***							
M0:one‐ratio	8534.934					*ω* = 0.138	Not allowed
M1: free‐ratio	8481.090	Mo versus M1	107.688	59	**0.000**	*ω* variation for each branch	Not allowed
***Site models***							
M1a (nearly neutral)	8257.877					*p*_*0*_ = 0.763, *p*_*1*_ = 0.237, *k*(kappa) = 2.031, *ω*_*0*_ = 0.067, *ω*_*1*_ = 1	Not allowed
M2a (positive selection)	8257.877	M2a versus M1a	0	2	1	*p*_*0*_ = 0.763, *p*_*1*_ = 0.185, *p*_*2*_ = 0.052, *k* = 2.031, *ω*_*0*_ = 0.067, *ω*_*1*_ = 1, *ω*_*2*_ = 1	
M7 (beta)	8156.904					*p* = 0.285, *q* = 1.246, *k* = 1.815	Not allowed
M8 (beta and omega)	8153.780	M8 versus M7	6.248	2	**0.044**	*p*_*0*_ = 0.933, *p*_*1*_ = 0.067, *p* = 0.354, *q* = 2.363, *k* = 1.836, *ω* = **1.025**	5 L(0.830),141 K(0.946)
M8a (beta)	8153.759	M8a versus M8	0.042	2	0.979	*p*_*0*_ = 0.927, *p*_*1*_ = 0.073, *p* = 0.357, *q* = 2.466, *k* = 1.835, *ω* = 1	
***Branch‐site model***							
**Ancestral Amphibia (branch a)**							
Alternative	8241.227	Alternative versus Null	3.58	1	0.059	*ω*_*0*_ = 0.060, *ω*_*1*_ = 1, *ω*_*2*_ = **82.700**	7P (0.817), 12Y (0.914), 13 N (0.878), 18I (0.960), 29S (0.989), 48E (0.983)
Null	8243.017					*ω*_*0*_ = 0.110, *ω*_*1*_ = 1, *ω*_*2*_ = 1	64D (0.970), 66T (0.974), 71D (0.919), 75K (0.940), 92I (0.934), 145T (0.869)
Caudata (branch e)							
Alternative	8257.877	Alternative versus Null	0	1	1	*ω*_*0*_ = 0.067, *ω*_*1*_ = 1, *ω*_*2*_ = 1	245E (0.988), 252K (0.986)
Null	8257.877					*ω*_*0*_ = 0.067, *ω*_*1*_ = 1, *ω*_*2*_ = 1	
Gymnophiona (branch b)							
Alternative	8257.877	Alternative versus Null	0	1	1	*ω*_*0*_ = 0.067, *ω*_*1*_ = 1, *ω*_*2*_ = 1	
Null	8257.877					*ω*_*0*_ = 0.067, *ω*_*1*_ = 1, *ω*_*2*_ = 1	
**Anura (branch c)**							
Alternative	8253.707	Alternative versus Null	6.702	1	**0.010**	*ω*_*0*_ = 0.068, *ω*_*1*_ = 1, *ω*_*2*_ = **221.480**	165A (0.874), 166Q (0.956)
Null	8257.058					*ω*_*0*_ = 0.067, *ω*_*1*_ = 1, *ω*_*2*_ = 1	
**Neobatrachia (branch d)**							
Alternative	8255.924	Alternative versus Null	2.586	1	0.108	*ω*_*0*_ = 0.067, *ω*_*1*_ = 1, *ω*_*2*_ = **13.313**	24T (0.971), 276H (0.863)
Null	8257.217					*ω*_*0*_ = 0.067, *ω*_*1*_ = 1, *ω*_*2*_ = 1	
***Clade model C***							
Ancestral Amphibia (branch a)	8157.713	M1a versus branch c	200.328	3	**0.000**	*ω*_*0*_ = 0.019, *p*_*0*_ = 0.571, *ω*_*1*_ = 1, *p*_*1*_ = 0.104, *ω*_*2*_ = 0.310, *ω*_*3*_ = 0.253, *p*_*2*_ = 0.325	
Caudata (branch e)	8156.332	M1a versus branch e	203.090	3	**0.000**	*ω*_*0*_ = 0.019, *p*_*0*_ = 0.575, *ω*_*1*_ = 1, *p*_*1*_ = 0.103, *ω*_*2*_ = 0.268, *ω*_*3*_ = 0.140, *p*_*2*_ = 0.322	
Gymnophiona (branch b)	8156.425	M1a versus branch e	202.904	3	**0.000**	*ω*_*0*_ = 0.019, *p*_*0*_ = 0.575, *ω*_*1*_ = 1, *p*_*1*_ = 0.100, *ω*_*2*_ = 0.271, *ω*_*3*_ = 0.171, *p*_*2*_ = 0.325	
Anura (branch c)	8155.714	M1a versus branch l	204.326	3	**0.000**	*ω*_*0*_ = 0.018, *p*_*0*_ = 0.580, *ω*_*1*_ = 1, *p*_*1*_ = 0.098, *ω*_*2*_ = 0.187, *ω*_*3*_ = 0.285, *p*_*2*_ = 0.322	
Neobatrachia (branch d)	8130.489	M1a versus branch l	254.776	3	**0.000**	*ω*_*0*_ = 0.018, *p*_*0*_ = 0.590, *ω*_*1*_ = 1, *p*_*1*_ = 0.008, *ω*_*2*_ = 0.173, *ω*_*3*_ = 0.528, *p*_*2*_ = 0.332	

^a^ The log‐likelihood difference between models compared.

^b^ The number of degrees of freedom equal to the difference in the numbers of free parameters between models.

^c^ Likelihood ratio test *p* values were adjusted for multiple testing with a Benjamini and Hochberg's procedure and threshold of 0.05.

^d^ Posterior probabilities of the BEB analysis with *p* > 0.8 considered as candidates of selection.

**TABLE 4:** Amino acid sites under positive selection with radical changes.

Gene	Site position	PAML			Datamonkey			TreeSAAP	
		M8 versus M7	Anura (branch c)	Neobatrachia (branch d)	FEL	MEME	SLAC	Radical changes in AA properties	Total
MYD88	5	√				√		Pα, Ns, Bl, RF, Pc, K0, F, αc, αn, Ra, Hp, Ht, Pt	13
	165		√			√		Pα, Pc, K0, αm, αn, Ht, Pt	7
TLR2	9				√	√		Pα, Ns, Br, RF, c, K0, pK′, Ca, h, Mv, Mw, V0, p, αn, Esm, Ra, Et, Pt	18
	19					√	√	Ns, Pβ, Br, RF, h, El, F, Pr, p, αc, αm, Ra, Hp, Et	14
	99	√					√	Pα, Ns, Pβ, Bl, Br, Pc, Ca, h, pHi, F, Mv, Mw, Hnc, V0, p, μ, Esm, Ra, Et, Pt	20
	165					√	√	Ns, Br, RF, Ca, h, pHi, El, F, Mv, Mw, Hnc, V0, Pr, p, αc, μ, Esm, Ra, Hp, Et	20
	172					√	√	Pα, Ns, Pβ, Br, RF, Pc, K0, pK′, h, El, F, Pr, p, αc, Ra, Hp, Ht, Et, Pt	19
	197				√		√	Pα, Ns, Pβ, RF, Pc, K0, pHi, El, F, V0, Pr, p, αc, αm, αn, Ht, Et, Pt	18
	209				√		√	Ns, Br, RF, Pc, pK′, h, Pr, p, αn, Ra, Et, Pt	12
	234					√	√	Pα, Pc, K0, pHi, F, αc, αm, αn, Esm, Ht, Pt	11
	274				√	√	√	Pα, K0, pHi, Ht, Et	5
	295				√	√	√	Ns, Pβ, Br, RF, Pc, pK′, h, El, F, Pr, p, αc, Ra, Hp, Et, Pt	16
	324					√	√	Pα, Ns, Pβ, Bl, Br, RF, Pc, K0, Ca, h, pHi, F, Mv, Mw, Hnc, V0, Pr, p, αc, μ, Ra, Ht, Et, Pt	24
	327					√	√	Pα, Ns, Pβ, Br, RF, pK′, h, pHi, El, F, Hnc, Pr, p, Ra, Hp, Ht, Et	17
	372				√	√		Pα, Pc, K0, Mv, Mw, V0, Pr, αc, Esm, Et, Pt	11
	378					√	√	Ns, Br, RF, Pc, c, K0, pK′, F, αc, μ, Esm, Ra, Hp, Pt	14
	403				√		√	Pβ, pK′	2
	474				√	√	√	Pα, Ns, Pc, pHi, p, αc, Ht, Pt	8
	520				√	√		Ns, Pβ, Bl, Br, RF, Pc, pK′, h, El, F, Pr, p, Ra, Hp, Ht, Et, Pt	17
	568					√	√	Ns, Br, RF, h, pHi, Hnc, p, αn, Esm, Et	10
	570				√	√		Ns, Pβ, Br, RF, h, pHi, El, F, Hnc, Pr, p, Ra, Hp, Et	14
	574				√	√		Ns, Br, RF, h, pHi, El, F, Pr, p, αc, αm, αn, Hp, Et, Pt	15
	637					√	√	Ns, Br, RF, c, pK′, Ca, h, pHi, Hnc, p, αn, μ, Esm, Ra, Et, Pt	16
	681					√	√	Pα, Ca, El, Mv, Mw, V0, μ, Ht	8
	740			√			√	Pα, pHi, Pr, αc, Pt	5
	773	√					√	Pα, Ns, Pβ, Br, RF, Pc, K0, pK′, h, El, F, Pr, p, αc, Ra, Hp, Ht, Et, Pt	19

*Note:* Physicochemical amino acid properties available in TreeSAAP are as following: Bl = Bulkiness, Br = Buriedness, c = Composition, Ca = Helical contact area, El = Long‐range non‐bonded energy, Esm = Short‐ and medium‐range non‐bonded energy, Et = Total non‐bonded energy,F = Mean r.m.s. fluctuational displacement, h = Hydropathy, Hnc = Normalized consensus hydrophobicity, Hp = Surrounding hydrophobicity, Ht = Thermodynamic transfer hydrophobicity, K0 = Compressibility, Mv = Molecular volume, Mw = Molecular weight, Ns = Average number of surrounding residues, *p* = Polarity, Pc = Coil tendency, pHi = Isoelectric point, pK′ = Equilibrium constant (ionization COOH), Pr = Polar requirement, Pt = Turn tendency, Pα = Alpha‐helical tendency, Pβ = Beta‐structure tendency, Ra = Solvent accessible reduction ratio, RF=Chromatographic index, V0 = Partial specific volume, αc = Power to be at the C‐terminal, αm = Power to be at the middle of the α‐helix, αn = Power to be at the N‐terminal, μ = Refractive index.

The branch‐site model was further used to test for positive selection in individual codons for lineages leading to the common ancestor of the Amphibia, Caudata, Gymnophiona, Anura, and Neobatrachia (Tables 2 and 3, branches a–e in Figures 4 and 5). The LRT tests showed evidence of significantly positive selection along the lineages leading to the ancestral Amphibia (*p* = 0.03), Anura (*p* = 0.001), Neobatrachia (*p* = 0.005) for TLR2, and Anura (*p* = 0.01) for MyD88, and a series of codons was detected under positive selection along these lineages with the BEB posterior probabilities ≥ 0.80 (Tables 2 and 3). Additionally, the lineage leading to the common ancestor of Neobatrachia (branch d in Figures 4 and 5) had a *ω* value of 13.313 for MyD88, and two positively selected sites were detected, although the LRT tests was not significant (Tables 2 and 3). We also found the ω value was higher than one (82.7) along the lineage leading to the common ancestor of Amphibia (branch a in Figure 5) for MyD88, and 14 codons were identified to be under positive selection (*p* value of 0.059) based on the LRT tests (Table 3). These positively selected sites were also shown to have undergone radical changes (Table 4) and they are involved in or near the LRR and TIR domains of TLR2, or death and TIR domains for MyD88 (Figures S5 and S6).

Clade model test revealed that TLR2 and MyD88 undergone significant selection divergence along lineages leading to the common ancestor of the Amphibia, Caudata, Gymnophiona, Anura, and Neobatrachia (Tables 2 and 3). Of these five clades examined, the ω value of the focal clade was not greater than one, indicating the action of purifying selection on these clades. The ω values of the focal clade were greater than those of the background clade for Amphibia (0.255 vs. 0.065), Anura (0.268 vs. 0.183), Neobatrachia (0.273 vs. 0.243) of TLR2, and Anura (0.285 vs. 0.187), Neobatrachia (0.528 vs. 0.173) of MyD88. The ω values of the focal clade were lower than those of the background clade for Amphibia (0.253 vs. 0.310), Caudata (0.140 vs. 0.268), Gymnophiona (0.171 vs. 0.271) of MyD88, and Gymnophiona (0.231 vs. 0.258) of TLR2 (Tables 2 and 3).

## 4 Discussion

Amphibians (frogs, toads, salamanders and newts), a highly specialized group of vertebrate, have long attracted the interests of evolutionary biologists and immunogeneticists alike (Babik et al. 2014; Niu et al. 2016; Bagheri and Zahmatkesh 2018; Tang et al. 2023). Amphibians must have faced new challenges of pathogens when transitioning from the aquatic to terrestrial environment and subsequently diversifying into various habitats (Fei et al. 2009; Grogan et al. 2018). TLRs are core components of the innate immune system in defense against invading pathogens in animals and crucial for triggering an appropriate immune response (Akira, Uematsu, and Takeuchi 2006; Babik et al. 2014; Grogan et al. 2018). However, little is known about the molecular evolution of the genes in the amphibian TLR signaling pathway. Here, we first determined the full‐length cDNA sequences and expression patterns of TLR2 and MyD88 in *Z. dennysi* , and examined the evolution of the two genes involved in TLR signaling in amphibians.

TLR2 and MyD88 were two crucial factors in the TLR signaling pathway (Zhang et al. 2014). Our results showed that the ZdTLR2 and ZdMyD88 had similar domains as its orthologs in other vertebrate species, suggesting similar functions (Bagheri and Zahmatkesh 2018; Tang et al. 2023). The extracellular LRR domain of ZdTLR2 contains nine LRR motifs that are different from other vertebrate species (Figure 1). The LRR domain is shown to be involved in pathogen recognition and the difference of the LRR number in different vertebrate TLR2 might reflect the need to acquire novel adaptations to recognize and respond to diverse pathogens presented by different environments for different vertebrate species (Zhang et al. 2014; Ishengoma and Agaba 2017). In addition, ZdTLR2 and ZdMyD88 were found to be expressed in all the organs/tissues examined (Figure 3), suggesting their important roles in the innate immune system for *Z. dennysi* as that in *Rana dybowskii* and three Japanese *Rana* frogs (Niu et al. 2016; Lau et al. 2018). The highly expression of both TLR2 and MyD88 genes were detected in the heart for *Z. dennysi* (Figure 3), which contradicts with the skin for TLR2 in three Japanese *Rana* frogs (Lau et al. 2018) and the lung for MyD88 in *X. tropicalis* (Tang et al. 2023). The different microhabitat types for *Z. dennysi* (arboreal), *Rana* frogs (terrestrial) and *X. tropicalis* (aquatic) (Fei et al. 2009), and different pathogenic microorganisms from different environments might contribute to the different expression patterns of the two genes in these frogs.

The TLR genes are highly conserved and have been regarded for a long time as functionally constrained in terrestrial vertebrates (Medzhitov and Janeway Jr. 1997; Roach et al. 2005; Barreiro et al. 2009; Mukherjee et al. 2009; Mukherjee, Ganguli, and Majumder 2014). The high amino acid similarities of the amphibian TLR2 and MyD88 sequences suggested that both genes were strongly conserved in all examined species of amphibians in the present study (Figures S5 and S6). The strong evolutionary conservation of amphibian TLR2 and MyD88 suggested their important function in the defense against pathogens for vertebrates (Babik et al. 2014; Bagheri and Zahmatkesh 2018; Tang et al. 2023). The analyses of the one‐ratio model (M0) also confirmed the strong functional constraints on amphibian TLR2 (*ω* = 0.215) and MyD88 (*ω* = 0.138) (Tables 2 and 3).

The sequence variation of TLRs is shown to reflect pathogen‐driven evolution (Tian et al. 2019; Xu et al. 2019; Zhang et al. 2022), although purifying selection was suggested for a long time to be the major force driving TLR evolution (Mukherjee et al. 2009). Previous studies have suggested the significance of pathogen interaction and positive selection pressure in the diversity of TLRs of vertebrate species (Xu et al. 2019; Velová et al. 2018; Priyam et al. 2018). The complex evolutionary history associated with the origin and diversification of amphibians from water to land posed great pathogenic challenges, making amphibians candidates of pathogen mediated selection on immune genes (Fei et al. 2009; Grogan et al. 2018; Zhang et al. 2022). Results obtained in this study provide evidence for recurrent positive selection acting on amphibian TLR2 and MyD88 genes (Table 2). It has shown that TLR2 and MyD88 are widely expressed across species and play essential roles in recognizing various PAMPs and inducing TLR‐mediated responses (Takeda, Kaisho, and Akira 2003; Zhang et al. 2014). More importantly, a series of significantly supported candidates of sites were identified to be under positive selection for amphibian TLR2 and MyD88 (Tables 2 and 3), and these codon changes had radical effects on their physicochemical properties (i.e., charge, polarity and volume) (Table 4). Generally, more radical amino acid substitutions have greater functional effects during evolution (Xu et al. 2019). In addition, these positive selected sites detected were found to be adjacent to or within the functional domains (e.g., LRR and TIR domains) of the two genes, and these corresponding amino acid changes might affect their secondary or tertiary conformation, which ultimately affected their function (Xu et al. 2019). Evidence of positive selection acting on TLR2 and MyD88 among different amphibian lineages was also detected (Tables 2 and 3), which was congruent with the evolutionary patterns of most vertebrate TLRs and MyD88 (Lau et al. 2018; Xu et al. 2019; Tang et al. 2023). The ω value of the lineage leading to the common ancestor of amphibians for TLR2 (branch a in Figure 4) was significantly higher than 1, which suggested that amphibians might have faced different pathogens during its transition from aquatic to terrestrial environment. It should be noted that strong signal of positive selection acting on TLR2 was identified along the lineage leading to the common ancestor of Anura (branch c in Figure 4) and modern frogs (Neobatrachia, branch d in Figure 4), further reflecting a faster adaptations to new pathogens as amphibians encountered novel habitat during their diversification (Grogan et al. 2018). Future studies should be conducted to confirm whether these changes indeed regulate the amphibian immune system during their transition from water to land.

## 5 Conclusions

In this study, we determined the full‐length cDNA sequences of TLR2 and MyD88 in *Z. dennysi* and explored their structure, expression patterns as well as the evolutionary patterns of TLR2 and MyD88 among amphibians. We found that recurrent positive selection has acted on TLR2 and MyD88 in amphibians and positively selected sites were mainly located at or close to function domains. Our results suggest that amphibians have adapted to different pathogenic microorganisms during their transition from the aquatic to terrestrial environment and diversification into various habitats. The present study will provide new insights into the evolutionary process and molecular basis underlying the immunological adaptation in vertebrates.

## Author Contributions

**Jie Zhang:** conceptualization (equal), data curation (equal), formal analysis (lead), funding acquisition (equal), methodology (equal), validation (equal), visualization (equal), writing – original draft (lead), writing – review and editing (equal). **Ruinan Zhao:** data curation (equal), investigation (lead), methodology (equal), resources (equal), validation (equal). **Hongyan Bi:** data curation (equal), investigation (lead), methodology (equal), resources (equal), validation (equal). **Jiaoying He:** data curation (equal), investigation (lead), methodology (equal), resources (equal), validation (equal). **Yang Guo:** data curation (equal), investigation (lead), methodology (equal), resources (equal), validation (equal). **Dian Liu:** data curation (equal), investigation (lead), methodology (equal), resources (equal), validation (equal). **Ganggang Yang:** conceptualization (equal), data curation (lead), methodology (equal), supervision (equal), validation (equal), writing – review and editing (equal). **Xiaohong Chen:** conceptualization (equal), data curation (lead), funding acquisition (equal), investigation (equal), methodology (equal), project administration (equal), resources (equal), supervision (equal), validation (equal), writing – original draft (equal), writing – review and editing (lead). **Zhuo Chen:** conceptualization (lead), data curation (lead), formal analysis (lead), funding acquisition (lead), investigation (lead), methodology (equal), project administration (equal), resources (lead), software (lead), supervision (lead), validation (equal), visualization (equal), writing – original draft (equal), writing – review and editing (lead).

## Conflicts of Interest

The authors declare no conflicts of interest.

## Supporting information


**Figure S1.** Nucleotide and deduced amino acid sequence of ZdTLR2. The LRR motifs are shown as single underlines (residues 50~494). The trans‐membrane structure is represented by a bold underline (residues 586~608), and the TIR domain is underlined by wavy lines (residues 636~781). Phosphorylation sites are marked with a square frame and N‐ glycosylation sites are marked with a circle. The alpha helix of the secondary structure is marked in red, and the beta helix is marked in green.

**Figure S2.** Nucleotide and deduced amino acid sequence of ZdMyD88. The Death domain is indicted by a dashed underline (residues 14–105), and the TIR domain is marked by wavy lines (residues 151–285). Phosphorylation sites are marked with square frame and N‐ glycosylation sites are marked with circle. The alpha helix of the secondary structure is show in red, and the beta helix in green.

**Figure S3.** Phylogenetic relationships of TLR2 in 41 representative vertebrates, constructed based on nucleotide sequences by MrBayes method. Numbers above the branches indicate the bootstrap support values for maximum likelihood inference and Bayesian posterior probability. Representative members are delimited by vertical lines to the right of the tree.

**Figure S4.** Phylogenetic relationships of MyD88 in 34 representative vertebrates, constructed based on nucleotide sequences by MrBayes method. Numbers above the branches indicate the bootstrap support values for maximum likelihood inference and Bayesian posterior probability. Representative members are delimited by vertical lines to the right of the tree.

**Figure S5.** Multiple sequence alignments of the representative amphibian TLR2. The positions of the LRR, trans‐membrane and TIR domains are indicated. Positively selected sites are shown in yellow colors. Information of the sequences used and the positively selected sites are shown in Tables 1 and 4.

**Figure S6.** Multiple sequence alignments of the representative amphibian MyD88. The positions of the death domain and TIR domain are indicated. Positively selected sites are shown in yellow colors. Information of the sequences used and the positively selected sites are shown in Tables 1 and 4.

**Table S1.** Primers used in this study.

**Table S2.** Positive selection sites for the TLR2 gene based on FEL analysis.

**Table S3.** Positive selection sites for the TLR2 gene based on MEME analysis.

**Table S4.** Positive selection sites for the TLR2 gene based on SLAC analysis.

**Table S5.** Positive selection sites for the MyD88 gene based on FEL analysis.

**Table S6.** Positive selection sites for the MyD88 gene based on MEME analysis.

## Data Availability

The data have been deposited in GenBank with the accession number PQ118635, PQ118636, and detailed information of the sequences used in this study can be found in Table 1. In addition, the sequences used for the pressure analysis were shown in Figures S5 and S6.
