# Supplementary material for: Positive Selection of TLR2 and MyD88 Genes Provides Insights Into the Molecular Basis of Immunological Adaptation in Amphibians
Source: Ecol Evol. 2024 Dec 16;14(12):e70723. doi: 10.1002/ece3.70723 (PMC11650749; doi:10.1002/ece3.70723)
Supplement: Supplementary file 1 — Figure S1. Nucleotide and deduced amino acid sequence of ZdTLR2. The LRR motifs are shown as single underlines (residues 50~494). The trans‐membrane structure is represented by a bold underline (residues 586~608), and the TIR domain is underlined by wavy lines (residues 636~781). Phosphorylation sites are marked with a square frame and N‐ glycosylation sites are marked with a circle. The alpha helix of the secondary structure is marked in red, and the beta helix is marked in green. [file ECE3-14-e70723-s006.pdf]

M V A Y A W S C

1 TGCACAATCACCAAGGGAGGACGCGGAGACACAAACGCTGCTCTAAATAGAAGATTGCAGCTGCTTCAACATCAGGATGGTTGCATACGCTTGGAGTTGT

C L I W S L V A V T H L L N G A E A C V C D D K N F C N C S S T H

101 TGCTTGATCTGGTCACTGGTGGCTGTAACACATTTACTGAATGGAGCTGAGGCCTGTGTTTGTGATGACAAGAATTTCTGTAACTGCTCTTCTACACATT

L T A I P P T L P K E L R W L D I S N N L I Q E I T D T D L Q P Y E

201 TGACTGCTATTCCTCCCACATTGCCAAAAGAACTGCGGTGGCTTGACATATCCAATAACTTAATTCAGGAGATTACAGATACAGACTTACAGCCATACGA

K L E V L L V N N N E I H T I S Q N A F Q P L R Y L E E L D I S Y

301 AAAGCTGGAAGTACTGCTCGTGAACAACAATGAAATCCATACCATCAGCCAAAATGCTTTTCAACCACTGCGTTATCTTGAAGAGTTGGATATATCTTAT

N K L T S M L P A W F G H L Q G L K R L N L L G N Q Y T S L G G T

401 AATAAACTAACCAGTATGTTGCCTGCTTGGTTTGGACACCTTCAAGGCTTGAAACGATTAAACCTGCTTGGTAACCAATATACTTCACTTGGTGGAACTC

P L F S T L S L L K D L K F G N A N F E V L H K H D F E G V L S L D

501 CCCTTTTTTCCACCCTGTCCTTGCTGAAGGATTTGAAATTTGGAAATGCTAACTTTGAAGTTTTACATAAGCATGACTTCGAAGGTGTGTTGAGCTTGGA

N L Y L N V S M L K Q Y T S N T L K T I K S V G H V T L T T N L T

601 TAACCTGTACTTAAATGTTTCAATGCTCAAGCAATATACAAGTAATACCCTAAAGACAATAAAATCTGTCGGCCATGTTACCCTTACTACAAATCTCACT

L L P E M I T D L S L S V I V L E I R N M S F F L H G D V E S F V

701 CTATTGCCAGAAATGATAACAGATCTTTCATTGTCGGTTATCGTATTAGAAATCAGAAACATGTCTTTCTTTCTGCATGGAGATGTAGAATCCTTTGTAG

A L N D T T A K V L M Y K S C L L T D Q S A A R L I E I I H T Y R N

801 CACTGAATGATACTACTGCTAAGGTCTTAATGTACAAAAGTTGCCTGCTTACTGACCAGAGTGCAGCACGACTTATAGAAATTATTCATACCTACAGAAA

V T D F V L D D C E L K G T G H G S A V L K D V N S S L S T I V I

901 TGTTACAGACTTTGTTTTGGATGATTGTGAACTAAAGGGGACAGGACATGGTTCTGCAGTTCTGAAGGATGTAAACTCCTCTCTTTCCACCATAGTGATC

K N L Y I P N F Y L F S D L S F A Y L V V R K I K S V T C I D S K

1001 AAGAATCTATACATTCCAAATTTTTATCTTTTTTCAGACTTAAGCTTTGCATATTTGGTTGTGCGGAAAATTAAAAGCGTTACATGTATTGATAGCAAAG

V F L M P C N F S R S F K M M E Y L D L S G N L L T D L L L E S T S

1101 TGTTTTTGATGCCTTGCAATTTTTCCAGATCCTTTAAGATGATGGAGTACCTTGACTTGAGTGGCAATCTCCTGACTGACCTGCTTTTGGAAAGCACATC

C F F D G F G A W P S L K T L N V S K N R L L S L P K A A E A L S

1201 TTGTTTTTTTGACGGATTTGGTGCTTGGCCCTCACTGAAAACTTTAAATGTGAGCAAAAACCGATTGCTCTCGTTACCAAAAGCTGCTGAAGCATTATCA

Y V P S L N S I D L S Q N S F G S S A L S S C T W P A N L K S L N

1301 TATGTGCCGTCTTTAAACAGCATTGATCTAAGTCAAAATAGTTTCGGAAGTTCAGCACTTTCTTCATGCACATGGCCTGCAAACCTCAAATCCTTAAATA

I S N C Q I R R F G K C I P G T L E R L D V S F N N L E E F V F S L

1401 TCTCAAATTGTCAGATCAGACGTTTTGGTAAATGCATTCCTGGAACTTTGGAGCGTTTGGATGTGAGTTTCAATAATCTTGAGGAATTTGTATTTTCTTT

P D L K E L Y I S D N R L T K L P A D A H L Y S L N L L I I R T N

1501 GCCAGATTTAAAAGAACTTTACATATCAGATAACAGATTAACTAAACTGCCAGCTGATGCCCATCTGTACAGTTTAAATCTTCTTATCATCCGAACAAAC

R L I D F F Q S D L N F F P N L T G L D G R N N N Y F C S C Q F V

1601 CGGCTCATTGACTTTTTCCAGTCTGACTTGAACTTCTTTCCAAATTTAACAGGATTGGATGGAAGGAACAATAACTATTTCTGCTCTTGTCAATTTGTGG

D F V S K N H E L L I G W S K D Y V C D S P T S V R G H Q I D K A N

1701 ATTTTGTTAGCAAAAATCATGAACTACTTATTGGTTGGTCAAAAGATTACGTTTGTGATTCTCCCACGTCTGTCAGAGGCCATCAAATTGACAAAGCAAA

L P L L M C H K T L I V T L T C I L L I L G I G I I L G L C Y Y F

1801 TCTTCCTCTCTTAATGTGCCACAAAACCTTGATTGTGACACTAACATGCATCCTCTTGATTTTAGGGATAGGTATTATTTTGGGACTCTGCTATTATTTT

H V L W Y V K M T W A W L K A K R R P L K V L D R E I C Y D A Y V

1901 CACGTTTTGTGGTACGTGAAGATGACCTGGGCCTGGCTGAAAGCAAAGAGGAGACCTTTAAAAGTACTGGACAGAGAAATCTGTTATGACGCATATGTAT

S Y S E R D S E W V E N M M L P L L E N G D P Q F K I C F H K R D F

2001 CATATAGCGAAAGAGATTCTGAATGGGTAGAAAATATGATGTTGCCATTGCTGGAAAATGGTGACCCTCAATTTAAAATATGCTTTCATAAGCGAGACTT

V P G K T I V D N I I D A M E T S Y K T L F I L S E H F V Q S E W

2101 TGTGCCTGGCAAAACGATCGTCGATAACATAATTGATGCCATGGAGACTAGCTACAAAACCCTTTTCATCTTGTCTGAACACTTTGTCCAGAGCGAATGG

C K Y E L E F S H F R L F D E N N D T A I L V I L E H I E R S T V

2201 TGCAAGTATGAGTTGGAGTTCTCACACTTTCGTCTTTTTGATGAAAATAATGACACAGCCATTTTGGTTATTCTGGAGCACATAGAAAGGTCGACTGTTC

P R R F A K L R K L M N T K T Y L K W P T E E E E Q E V F W S N L R

2301 CAAGAAGATTTGCCAAACTGCGCAAACTCATGAACACAAAAACCTATCTTAAATGGCCAACAGAGGAAGAGGAGCAAGAAGTCTTCTGGTCCAACCTGAG

A A L Q P E D H L P V –

2401 GGCAGCCCTGCAGCCAGAAGACCACTTACCAGTATAGAGGAAAACATGATGT
