# Supplementary material for: Positive Selection of TLR2 and MyD88 Genes Provides Insights Into the Molecular Basis of Immunological Adaptation in Amphibians
Source: Ecol Evol. 2024 Dec 16;14(12):e70723. doi: 10.1002/ece3.70723 (PMC11650749; doi:10.1002/ece3.70723)
Supplement: Supplementary file 2 — Figure S2. Nucleotide and deduced amino acid sequence of ZdMyD88. The Death domain is indicted by a dashed underline (residues 14–105), and the TIR domain is marked by wavy lines (residues 151–285). Phosphorylation sites are marked with square frame and N‐ glycosylation sites are marked with circle. The alpha helix of the secondary structure is show in red, and the beta helix in green. [file ECE3-14-e70723-s010.pdf]

```
                                                                              M A G V P S P V Y L D Y N S I
1    TGAAGACCCTAAAGCTTTTTCAAAAGGATTACTAGTCGGTCAGAGGATAGAACTATGGCTGGTGTACCAAGTCCGGTATATCTGGACTACAACTCAATTC
     P L I A L N F T T R Q K L S L Y L N P E A V T A S D W T H L A E E M
101  CACTCATTGCTTTGAACTTTACTACCAGGCAGAAGTTGTCTTTATATTTGAACCCCGAGGCAGTGACGGCTTCAGACTGGACCCATCTTGCAGAAGAAAT
     E Y N Y L E I R N F Q R F P D P T S S L L D D W Q K K H S K A T V
201  GGAATATAACTATCTAGAGATAAGGAATTTTCAGAGATTTCCTGACCCAACATCCTCACTTCTGGATGACTGGCAGAAAAAACATTCAAAAGCAACTGTT
     G E L L N L L Q K I E R H D I L T D L A T L I D G D C Q K H L R R
301  GGGGAACTGCTGAACCTGCTGCAGAAGATAGAAAGGCATGATATTCTCACAGATCTAGCTACTTTAATAGATGGGGACTGTCAGAAGCATTTAAGAAGGA
     S K P S G K P P P V Q D E T V D S S G S Q C L T T G D D P S G H L P
401  GCAAACCGTCCGGGAAACCACCACCAGTACAAGATGAAACCGTGGACAGTAGTGGAAGTCAGTGTTTGACCACGGGTGATGACCCTTCAGGACATCTGCC
     E Q F D A F I C Y C A Q D I S F V Q E M I S R L E Q T D H N L K L
501  CGAGCAGTTTGATGCCTTCATCTGCTATTGTGCCCAGGATATAAGTTTTGTGCAGGAAATGATCAGTCGGCTGGAGCAGACGGACCATAATCTGAAGCTG
     C V F D R D V L P G T C L W S I T S E L I E K R C R K M V V V I S
601  TGTGTGTTTGATAGAGACGTCCTCCCCGGGACATGTCTGTGGTCTATAACAAGTGAACTTATAGAAAAAAGGTGTAGGAAGATGGTGGTGGTTATATCTG
     D D Y L D S N E C D F Q T K F A L S L G P G A R E R R L I P V T Y K
701  ATGATTATTTGGACAGCAATGAGTGTGATTTCCAGACAAAATTTGCTCTCAGCCTCGGACCAGGCGCTCGTGAGAGAAGACTCATTCCAGTGACATACAA
     A M K R P F P T I L R F I T A C D Y T K P N I Q G W F W D R L A K
801  AGCCATGAAGAGACCTTTTCCCACCATCCTCCGGTTCATTACAGCGTGTGATTATACAAAACCCAACATACAAGGATGGTTTTGGGACAGGCTTGCTAAA
     A L K R –
901  GCTCTAAAGAGATGAGTGATAAAATCTTGGAACTTCAAGAGGTTATCTTAGGGCCTCACTAGGATGATCTTGGGAATACTTTCGTTACTTGCTTTCCCTA

1001 CTTCCTCTTCTTGTGAGGAGCTGCTCAAACTTAAAGTTCTCAAATACAGGTTTATGTATATATACGGAGTGGGGCAGTTGATGTGATACTGCCGTGAATG

1101 TTACAAATAAACTCATGGCTTTCGT
```
