# Supplementary figures and images for: Positive Selection of TLR2 and MyD88 Genes Provides Insights Into the Molecular Basis of Immunological Adaptation in Amphibians
Source: Ecol Evol. 2024 Dec 16;14(12):e70723. doi: 10.1002/ece3.70723 (PMC11650749; doi:10.1002/ece3.70723)

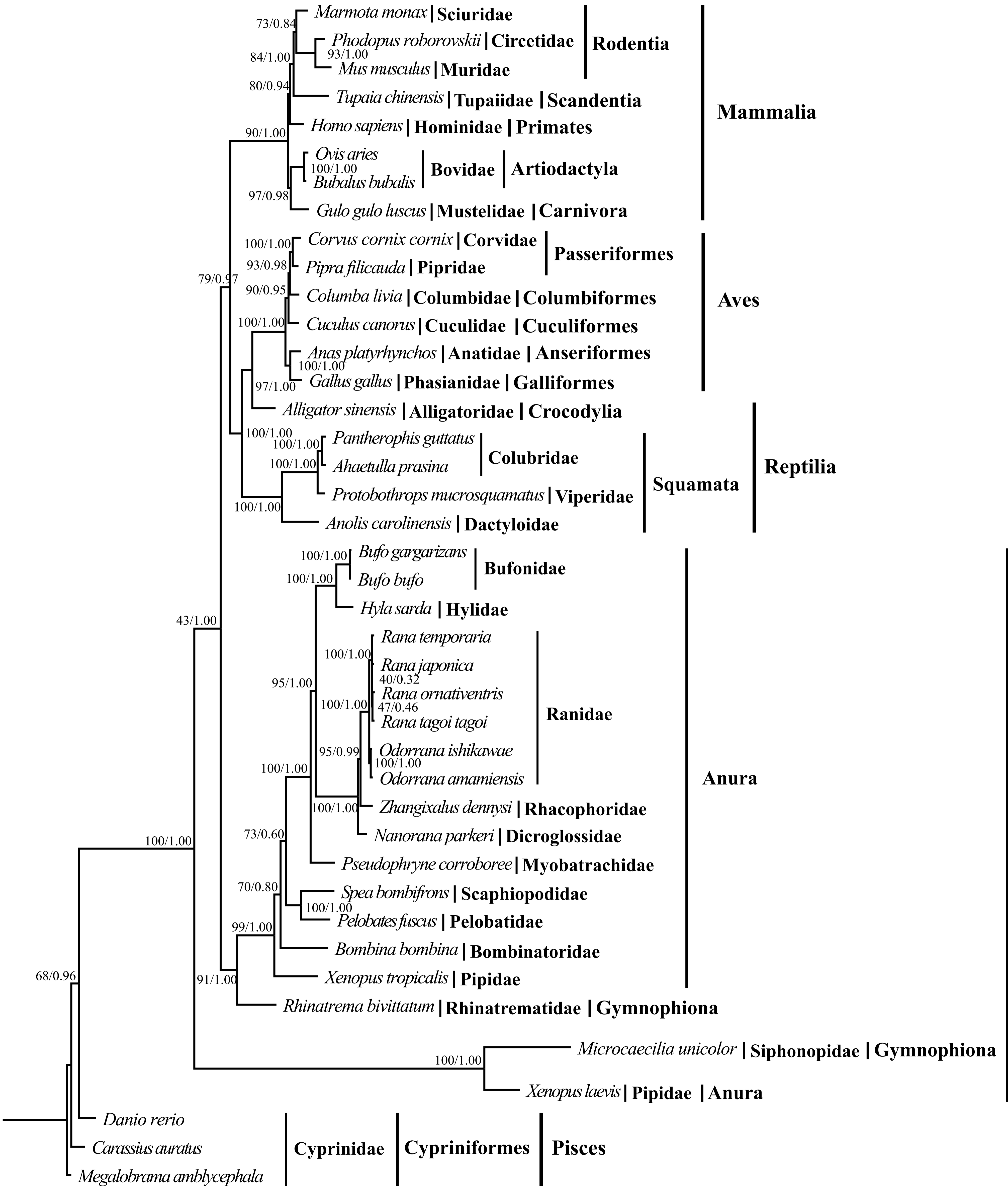

Supplement: Supplementary file 3 — Figure S3. Phylogenetic relationships of TLR2 in 41 representative vertebrates, constructed based on nucleotide sequences by MrBayes method. Numbers above the branches indicate the bootstrap support values for maximum likelihood inference and Bayesian posterior probability. Representative members are delimited by vertical lines to the right of the tree. [file ECE3-14-e70723-s001.pdf]

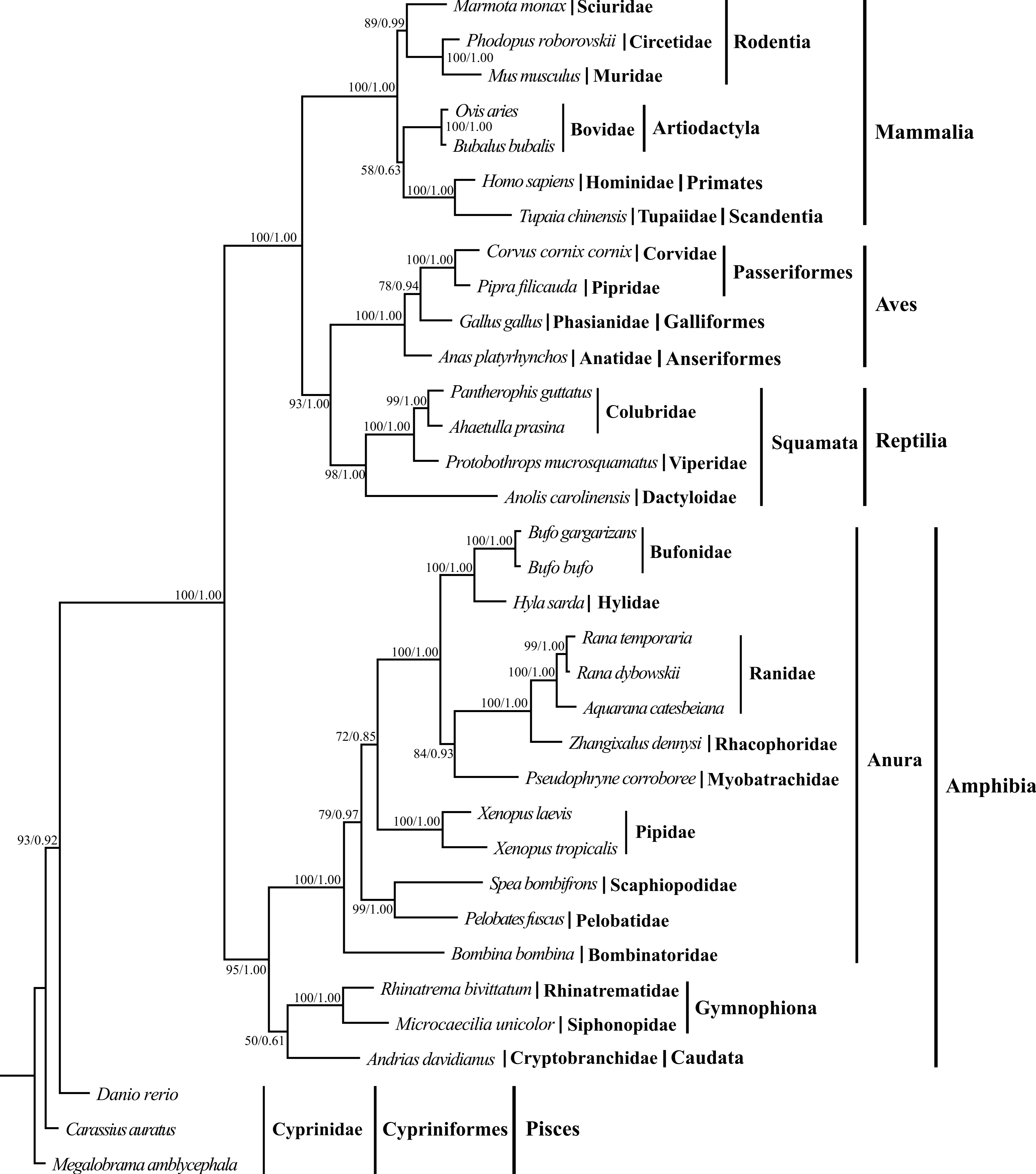

Supplement: Supplementary file 4 — Figure S4. Phylogenetic relationships of MyD88 in 34 representative vertebrates, constructed based on nucleotide sequences by MrBayes method. Numbers above the branches indicate the bootstrap support values for maximum likelihood inference and Bayesian posterior probability. Representative members are delimited by vertical lines to the right of the tree. [file ECE3-14-e70723-s008.pdf]
