# Supplementary material for: Positive Selection of TLR2 and MyD88 Genes Provides Insights Into the Molecular Basis of Immunological Adaptation in Amphibians
Source: Ecol Evol. 2024 Dec 16;14(12):e70723. doi: 10.1002/ece3.70723 (PMC11650749; doi:10.1002/ece3.70723)
Supplement: Supplementary file 5 — Figure S5. Multiple sequence alignments of the representative amphibian TLR2. The positions of the LRR, trans‐membrane and TIR domains are indicated. Positively selected sites are shown in yellow colors. Information of the sequences used and the positively selected sites are shown in Tables 1 and 4. [file ECE3-14-e70723-s012.pdf]

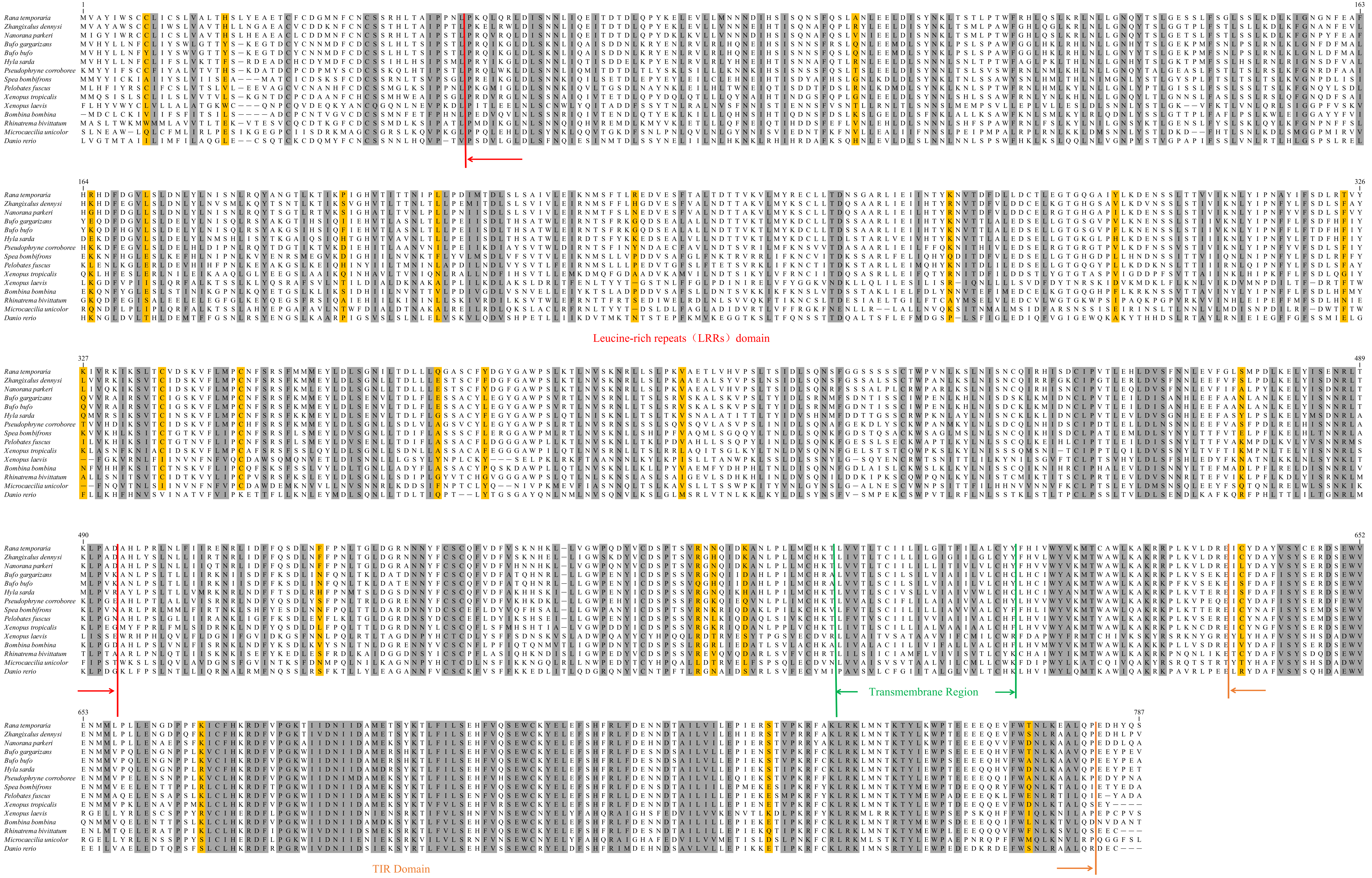

1
163
164
326
327
489
490
652
653
787
Rana temporaria
Zhangixalus dennysi
Nanorana parkeri
Bufo gargarizans
Bufo bufo
Hyla sarda
Pseudophryne corroboree
Spea bombifrons
Pelobates fuscus
Xenopus tropicalis
Xenopus laevis
Bombina bombina
Rhinatrema bivittatum
Microcaecilia unicolor
Danio rerio
Leucine-rich repeats（LRRs）domain
Transmembrane Region
TIR Domain
