# Supplementary material for: Positive Selection of TLR2 and MyD88 Genes Provides Insights Into the Molecular Basis of Immunological Adaptation in Amphibians
Source: Ecol Evol. 2024 Dec 16;14(12):e70723. doi: 10.1002/ece3.70723 (PMC11650749; doi:10.1002/ece3.70723)
Supplement: Supplementary file 7 — Table S1. Primers used in this study. [file ECE3-14-e70723-s007.docx]

Table S1 Primers used in this study

| Primer | Sequence（5’→3’） | Application | Fragment size (bp) |
| --- | --- | --- | --- |
| TLR2-3’-Out | TCAGTCAGGAGATTGCCACTCAA | first round of TLR2 3' end | 1281 |
| TLR2-3’-In | GGCCTCTGACAGACGTGGGAGAA | second round of TLR2 3' end | 694 |
| TLR2-5’-Out | TGTTACCCTTACTACAAATCTCAC | first round of TLR2 2' end | 699 |
| TLR2-5’-In | CACTGCGTTATCTTGAAGAGTTG | second round of TLR2 2' end | 388 |
| TLR2-F | CGTGAACAACAATGAAATCCATACC | TLR2 intermediate fragment | 1584 |
| TLR2-R | TGAAAATAATAGCAGAGTCCCAAAA | TLR2 intermediate fragment |  |
| 3’RACE Oligo（T）-Adaptor | CTGATCTAGAGGTACCGGATCC(T)_14_ | 3' RACE reverse transcription | / |
| 3’RACE Adaptor | CTGATCTAGAGGTACCGGATCC | 3' universal primer | / |
| 5’RACE Oligo（T）-Adaptor | GACTCGAGTCGACATCGA(T)_17_ | 5' First round universal primer | / |
| 5’RACE Adaptor | GACTCGAGTCGACATCG | 5' second round universal primer | / |
| TLR2-RT-F | GCCAAACTGCGCAAACTCA | TLR2 qRT-RCR | 121 |
| TLR2-RT-R | CTGGTAAGTGGTCTTCTGGCT | TLR2 qRT-RCR |  |
| MyD88-RT-F | ACCGTAATGGCAATAGGCTGAC | MyD88 qRT-RCR | 132 |
| MyD88-RT-R | AATCGCACCAAACAACTGACTC | MyD88 qRT-RCR |  |
| β- actin -RT-F | AACACCCAACCCTTCTCACA | β- actin qRT-RCR | 117 |
| β- actin -RT-R | CCGCCTGGATAGCCACATA | β- actin qRT-RCR |  |
