# Supplementary material for: Positive Selection of TLR2 and MyD88 Genes Provides Insights Into the Molecular Basis of Immunological Adaptation in Amphibians
Source: Ecol Evol. 2024 Dec 16;14(12):e70723. doi: 10.1002/ece3.70723 (PMC11650749; doi:10.1002/ece3.70723)
Supplement: Supplementary file 8 — Table S2. Positive selection sites for the TLR2 gene based on FEL analysis. [file ECE3-14-e70723-s004.docx]

Table S2. Positive selection sites for the TLR2 gene based on FEL analysis.

| NO. | Site | α | β | α=β | p-value | Estimate of parameters(ω) |
| --- | --- | --- | --- | --- | --- | --- |
| 1 | 9 | 0 | 0.515 | 0.408 | 0.0376 | 10 |
| 2 | 18 | 0.089 | 0.516 | 0.363 | 0.0809 | 5.78 |
| 3 | 26 | 0 | 0.442 | 0.348 | 0.1089 | 10 |
| 4 | 86 | 0 | 0.464 | 0.384 | 0.1073 | 10 |
| 5 | 197 | 0 | 2.11 | 1.512 | 0.0597 | 10 |
| 6 | 209 | 0 | 1.105 | 0.63 | 0.1354 | 10 |
| 7 | 274 | 0 | 0.623 | 0.385 | 0.0792 | 10 |
| 8 | 295 | 0 | 3.694 | 1.524 | 0.0107 | 10 |
| 9 | 372 | 0 | 0.558 | 0.379 | 0.0414 | 10 |
| 10 | 403 | 0 | 0.234 | 0.171 | 0.1041 | 10 |
| 11 | 474 | 0 | 0.78 | 0.623 | 0.0379 | 10 |
| 12 | 477 | 0.22 | 1.317 | 0.905 | 0.1826 | 6 |
| 13 | 520 | 0.381 | 1.75 | 1.294 | 0.2 | 4.6 |
| 14 | 526 | 0 | 1.412 | 0.999 | 0.111 | 10 |
| 15 | 570 | 0 | 0.67 | 0.528 | 0.0193 | 10 |
| 16 | 574 | 0 | 0.502 | 0.426 | 0.1192 | 10 |
