# Supplementary material for: Positive Selection of TLR2 and MyD88 Genes Provides Insights Into the Molecular Basis of Immunological Adaptation in Amphibians
Source: Ecol Evol. 2024 Dec 16;14(12):e70723. doi: 10.1002/ece3.70723 (PMC11650749; doi:10.1002/ece3.70723)
Supplement: Supplementary file 9 — Table S3. Positive selection sites for the TLR2 gene based on MEME analysis. [file ECE3-14-e70723-s011.docx]

Table S3. Positive selection sites for the TLR2 gene based on MEME analysis.

| NO. | Site | α | β- | p- | β+ | p+ | p-value |
| --- | --- | --- | --- | --- | --- | --- | --- |
| 1 | 5 | 4.96 | 1.9 | 0 | 1117.55 | 1 | 0.09 |
| 2 | 9 | 0 | 0 | 0 | 13.76 | 1 | 0 |
| 3 | 19 | 0 | 0 | 0 | 10.66 | 1 | 0.06 |
| 4 | 52 | 0 | 0 | 0 | 19.51 | 1 | 0.01 |
| 5 | 140 | 0.35 | 0.35 | 0 | 22.31 | 1 | 0.09 |
| 6 | 146 | 2.79 | 0.2 | 0 | 19.81 | 1 | 0.08 |
| 7 | 165 | 0.97 | 0 | 0 | 29.76 | 1 | 0.02 |
| 8 | 172 | 1.88 | 0.31 | 0 | 79.64 | 1 | 0.03 |
| 9 | 186 | 3.45 | 1.91 | 0 | 80.79 | 1 | 0.08 |
| 10 | 233 | 0.37 | 0.31 | 0 | 40998.46 | 1 | 0 |
| 11 | 234 | 3.16 | 0.54 | 0 | 23.96 | 1 | 0.1 |
| 12 | 238 | 1.43 | 0.8 | 0 | 4286.34 | 1 | 0.03 |
| 13 | 274 | 0 | 0 | 0 | 4.54 | 1 | 0.09 |
| 14 | 295 | 0 | 0 | 0 | 27.88 | 1 | 0.02 |
| 15 | 324 | 1.88 | 0.97 | 0 | 56.31 | 1 | 0.09 |
| 16 | 327 | 0.72 | 0.72 | 0 | 92.65 | 1 | 0.01 |
| 17 | 372 | 0 | 0 | 0 | 3.09 | 1 | 0.03 |
| 18 | 378 | 1.24 | 0.78 | 0 | 25.06 | 1 | 0.09 |
| 19 | 408 | 2.69 | 1.27 | 0 | 473.13 | 1 | 0.04 |
| 20 | 474 | 0 | 0 | 0 | 5.54 | 1 | 0.06 |
| 21 | 520 | 1.39 | 0.28 | 0 | 26.86 | 1 | 0.09 |
| 22 | 522 | 1.73 | 0 | 0 | 23.74 | 1 | 0.08 |
| 23 | 531 | 0.99 | 0.99 | 0 | 59.33 | 1 | 0.09 |
| 24 | 568 | 2.23 | 0 | 0 | 502.4 | 1 | 0 |
| 25 | 570 | 0 | 0 | 0 | 6.33 | 1 | 0.03 |
| 26 | 574 | 0 | 0 | 0 | 15.06 | 1 | 0.06 |
| 27 | 637 | 6.77 | 0.56 | 0 | 84.96 | 1 | 0.05 |
| 28 | 681 | 0 | 0 | 0 | 17.96 | 1 | 0.09 |
