# Supplementary material for: Positive Selection of TLR2 and MyD88 Genes Provides Insights Into the Molecular Basis of Immunological Adaptation in Amphibians
Source: Ecol Evol. 2024 Dec 16;14(12):e70723. doi: 10.1002/ece3.70723 (PMC11650749; doi:10.1002/ece3.70723)
Supplement: Supplementary file 10 — Table S4. Positive selection sites for the TLR2 gene based on SLAC analysis. [file ECE3-14-e70723-s005.docx]

Table S4. Positive selection sites for the TLR2 gene based on SLAC analysis.

| NO. | Site | ES | EN | S | N | P[S] | dS | dN | dN-dS | P [dN/dS > 1] |
| --- | --- | --- | --- | --- | --- | --- | --- | --- | --- | --- |
| 1 | 2 | 0.886343 | 2.113593 | 2.333333 | 30.66667 | 0.295454 | 2.632539 | 14.50926 | 3.41E-07 | 0.002315 |
| 2 | 3 | 0.764909 | 2.217711 | 2.666667 | 20.33333 | 0.256455 | 3.486252 | 9.168611 | 1.63E-07 | 0.095928 |
| 3 | 7 | 0.794962 | 2.204978 | 1.833333 | 17.16667 | 0.264993 | 2.306189 | 7.785415 | 1.57E-07 | 0.075979 |
| 4 | 10 | 0.663887 | 2.280836 | 4.083333 | 29.91667 | 0.22545 | 6.150646 | 13.11653 | 2E-07 | 0.099212 |
| 5 | 14 | 0.6906 | 2.259618 | 2.833333 | 35.16667 | 0.234084 | 4.102712 | 15.5631 | 3.29E-07 | 0.011077 |
| 6 | 15 | 0.676711 | 2.305939 | 3.666667 | 33.33333 | 0.226882 | 5.418368 | 14.45542 | 2.59E-07 | 0.043403 |
| 7 | 16 | 0.897314 | 2.102648 | 2.833333 | 23.16667 | 0.299108 | 3.157571 | 11.01785 | 2.26E-07 | 0.023257 |
| 8 | 19 | 0.794769 | 2.205134 | 0.5 | 22.5 | 0.264932 | 0.629113 | 10.20346 | 2.75E-07 | 0.004334 |
| 9 | 20 | 0.865844 | 2.089278 | 6.916667 | 31.08333 | 0.292998 | 7.988348 | 14.87755 | 1.98E-07 | 0.089758 |
| 10 | 22 | 0.794401 | 2.132781 | 0.5 | 19.5 | 0.271388 | 0.629405 | 9.142991 | 2.44E-07 | 0.008401 |
| 11 | 24 | 0.896827 | 2.103033 | 2.5 | 24.5 | 0.298956 | 2.787605 | 11.64984 | 2.55E-07 | 0.012982 |
| 12 | 27 | 0.79488 | 2.12407 | 4 | 28 | 0.272317 | 5.032205 | 13.18224 | 2.34E-07 | 0.040028 |
| 13 | 29 | 0.79501 | 2.124115 | 2 | 27 | 0.272345 | 2.515691 | 12.71118 | 2.93E-07 | 0.006807 |
| 14 | 35 | 0.795042 | 2.124048 | 2.5 | 28.5 | 0.272359 | 3.14449 | 13.41778 | 2.95E-07 | 0.010252 |
| 15 | 40 | 0.712241 | 2.239929 | 2.333333 | 24.66667 | 0.24126 | 3.276044 | 11.01225 | 2.22E-07 | 0.04423 |
| 16 | 42 | 0.663949 | 2.280816 | 4.166667 | 36.83333 | 0.225468 | 6.27558 | 16.14919 | 2.83E-07 | 0.037642 |
| 17 | 43 | 0.89685 | 2.103086 | 2 | 16 | 0.298956 | 2.230028 | 7.607867 | 1.54E-07 | 0.061052 |
| 18 | 44 | 0.896781 | 2.10306 | 0.5 | 26.5 | 0.298943 | 0.55755 | 12.60069 | 3.46E-07 | 0.000462 |
| 19 | 46 | 0.897326 | 2.102627 | 3.5 | 30.5 | 0.299113 | 3.900477 | 14.50566 | 3.04E-07 | 0.007638 |
| 20 | 49 | 0.729581 | 2.228541 | 2.166667 | 35.83333 | 0.246636 | 2.969743 | 16.07928 | 3.77E-07 | 0.002927 |
| 21 | 50 | 0.765198 | 2.198397 | 6 | 33 | 0.258199 | 7.841109 | 15.01094 | 2.06E-07 | 0.091186 |
| 22 | 53 | 0.756105 | 2.207465 | 5.25 | 30.75 | 0.255133 | 6.943481 | 13.93 | 2.01E-07 | 0.093048 |
| 23 | 54 | 0.526118 | 2.373782 | 1.833333 | 25.16667 | 0.181426 | 3.484643 | 10.60193 | 2.04E-07 | 0.095943 |
| 24 | 55 | 0.760351 | 2.139199 | 3.666667 | 29.33333 | 0.262231 | 4.822334 | 13.7123 | 2.55E-07 | 0.033274 |
| 25 | 56 | 0.793658 | 2.180354 | 2.083333 | 37.91667 | 0.266864 | 2.624976 | 17.39015 | 4.24E-07 | 0.000642 |
| 26 | 58 | 0.754255 | 2.22595 | 3.333333 | 35.66667 | 0.253088 | 4.419373 | 16.02312 | 3.33E-07 | 0.009318 |
| 27 | 62 | 0.824061 | 2.129354 | 3 | 30 | 0.27902 | 3.640509 | 14.08878 | 3E-07 | 0.008381 |
| 28 | 64 | 0.397937 | 2.476018 | 0 | 27 | 0.138463 | 0 | 10.9046 | 3.13E-07 | 0.017881 |
| 29 | 71 | 0.788657 | 2.140033 | 4.083333 | 33.91667 | 0.269287 | 5.177578 | 15.84867 | 3.06E-07 | 0.014473 |
| 30 | 77 | 0.756165 | 2.20736 | 4.583333 | 36.41667 | 0.255157 | 6.061288 | 16.49784 | 3E-07 | 0.022717 |
| 31 | 80 | 0.756208 | 2.207345 | 4.75 | 32.25 | 0.25517 | 6.281336 | 14.61031 | 2.39E-07 | 0.052723 |
| 32 | 94 | 0.897485 | 2.078197 | 2 | 27 | 0.301606 | 2.228449 | 12.99203 | 3.09E-07 | 0.002688 |
| 33 | 95 | 0.818304 | 2.147518 | 3 | 28 | 0.275911 | 3.666121 | 13.03831 | 2.69E-07 | 0.014817 |
| 34 | 98 | 0.756153 | 2.207417 | 3.333333 | 28.66667 | 0.255149 | 4.40828 | 12.98652 | 2.46E-07 | 0.035017 |
| 35 | 99 | 0.7934 | 2.194233 | 2.5 | 27.5 | 0.265561 | 3.150997 | 12.53286 | 2.69E-07 | 0.015679 |
| 36 | 101 | 0.886591 | 2.113345 | 2 | 30 | 0.295537 | 2.255831 | 14.19551 | 3.43E-07 | 0.001377 |
| 37 | 102 | 0.397917 | 2.552379 | 0.333333 | 26.66667 | 0.134873 | 0.837697 | 10.44777 | 2.76E-07 | 0.048075 |
| 38 | 104 | 0.729427 | 2.2287 | 3.166667 | 36.83333 | 0.246584 | 4.341304 | 16.52682 | 3.5E-07 | 0.007463 |
| 39 | 111 | 0.83931 | 2.124189 | 4.083333 | 37.91667 | 0.283216 | 4.865109 | 17.84995 | 3.73E-07 | 0.003604 |
| 40 | 114 | 0.66584 | 2.314401 | 2.75 | 33.25 | 0.223418 | 4.130119 | 14.36657 | 2.94E-07 | 0.021272 |
| 41 | 116 | 0.897204 | 2.10264 | 4.25 | 28.75 | 0.299084 | 4.73694 | 13.67329 | 2.57E-07 | 0.022045 |
| 42 | 117 | 0.897397 | 2.10257 | 2.5 | 24.5 | 0.299136 | 2.785836 | 11.65241 | 2.55E-07 | 0.01292 |
| 43 | 118 | 0.55783 | 2.404347 | 0.25 | 40.75 | 0.188318 | 0.448165 | 16.94847 | 4.74E-07 | 0.000651 |
| 44 | 119 | 0.818528 | 2.147347 | 0.333333 | 27.66667 | 0.275982 | 0.407235 | 12.88412 | 3.58E-07 | 0.000539 |
| 45 | 122 | 0.886631 | 2.113308 | 0.916667 | 32.08333 | 0.29555 | 1.033876 | 15.18157 | 4.06E-07 | 0.00013 |
| 46 | 125 | 0.663835 | 2.280889 | 3.083333 | 38.91667 | 0.225432 | 4.644731 | 17.06206 | 3.57E-07 | 0.009552 |
| 47 | 128 | 0.788677 | 2.140004 | 4.25 | 36.75 | 0.269294 | 5.388773 | 17.17286 | 3.38E-07 | 0.009853 |
| 48 | 130 | 0.815149 | 2.148432 | 4.833333 | 32.16667 | 0.275055 | 5.929387 | 14.97216 | 2.6E-07 | 0.032277 |
| 49 | 131 | 0.88646 | 2.113402 | 5 | 33 | 0.2955 | 5.640414 | 15.61463 | 2.86E-07 | 0.015854 |
| 50 | 132 | 0.863082 | 2.1369 | 4.333333 | 23.66667 | 0.287696 | 5.020764 | 11.07523 | 1.74E-07 | 0.088923 |
| 51 | 138 | 0.886698 | 2.113167 | 2.583333 | 30.41667 | 0.295579 | 2.91343 | 14.39388 | 3.3E-07 | 0.003264 |
| 52 | 139 | 0.863318 | 2.136649 | 2.333333 | 28.66667 | 0.287776 | 2.702751 | 13.41664 | 3.08E-07 | 0.005081 |
| 53 | 143 | 0.886439 | 2.113541 | 5.666667 | 28.33333 | 0.295482 | 6.392621 | 13.40562 | 2.01E-07 | 0.070594 |
| 54 | 147 | 0.75623 | 2.207358 | 3.833333 | 33.16667 | 0.255174 | 5.069005 | 15.0255 | 2.86E-07 | 0.02145 |
| 55 | 150 | 0.75617 | 2.207355 | 5.166667 | 37.83333 | 0.255159 | 6.83268 | 17.13967 | 2.96E-07 | 0.026804 |
| 56 | 152 | 0.794929 | 2.20497 | 2.25 | 19.75 | 0.264985 | 2.830443 | 8.957039 | 1.76E-07 | 0.065216 |
| 57 | 153 | 0.663987 | 2.28074 | 3.25 | 37.75 | 0.225483 | 4.894672 | 16.55164 | 3.35E-07 | 0.014871 |
| 58 | 155 | 0.851536 | 2.138575 | 1.5 | 25.5 | 0.284784 | 1.761523 | 11.92383 | 2.92E-07 | 0.004648 |
| 59 | 156 | 0.738242 | 2.212078 | 3.666667 | 35.33333 | 0.250224 | 4.966754 | 15.97291 | 3.16E-07 | 0.014797 |
| 60 | 158 | 0.87299 | 2.126913 | 3.416667 | 31.58333 | 0.291006 | 3.913752 | 14.84938 | 3.14E-07 | 0.007032 |
| 61 | 160 | 0.81832 | 2.147523 | 4.083333 | 28.91667 | 0.275915 | 4.989899 | 13.46512 | 2.43E-07 | 0.033376 |
| 62 | 162 | 0.893773 | 2.106161 | 4.333333 | 25.66667 | 0.297931 | 4.848361 | 12.18647 | 2.11E-07 | 0.047893 |
| 63 | 163 | 0.80997 | 2.145221 | 3.75 | 26.25 | 0.274084 | 4.629801 | 12.2365 | 2.18E-07 | 0.047666 |
| 64 | 164 | 0.794849 | 2.205083 | 1.666667 | 21.33333 | 0.264956 | 2.096833 | 9.674619 | 2.18E-07 | 0.026269 |
| 65 | 165 | 0.606311 | 2.362467 | 0.666667 | 22.33333 | 0.204229 | 1.099546 | 9.453393 | 2.4E-07 | 0.025789 |
| 66 | 166 | 0.79489 | 2.205042 | 0 | 18 | 0.264969 | 0 | 8.163111 | 2.34E-07 | 0.003922 |
| 67 | 168 | 0.756649 | 2.197785 | 2.5 | 28.5 | 0.256106 | 3.304043 | 12.9676 | 2.77E-07 | 0.016477 |
| 68 | 169 | 0.680484 | 2.305118 | 2 | 22 | 0.227922 | 2.939085 | 9.54398 | 1.9E-07 | 0.064696 |
| 69 | 170 | 0.774304 | 2.190354 | 2.833333 | 30.16667 | 0.261178 | 3.6592 | 13.77251 | 2.9E-07 | 0.012829 |
| 70 | 171 | 0.676605 | 2.306004 | 4.833333 | 34.16667 | 0.22685 | 7.143505 | 14.81639 | 2.2E-07 | 0.08601 |
| 71 | 172 | 0.663908 | 2.280856 | 1.416667 | 29.58333 | 0.225454 | 2.133831 | 12.97028 | 3.11E-07 | 0.00961 |
| 72 | 174 | 0.752591 | 2.18072 | 2.583333 | 37.41667 | 0.256567 | 3.432588 | 17.15794 | 3.94E-07 | 0.002438 |
| 73 | 177 | 0.815313 | 2.148265 | 4.666667 | 29.33333 | 0.275111 | 5.72377 | 13.65443 | 2.28E-07 | 0.050495 |
| 74 | 179 | 0.78847 | 2.140185 | 4.583333 | 34.41667 | 0.269226 | 5.812943 | 16.08116 | 2.95E-07 | 0.021271 |
| 75 | 180 | 0.7945 | 2.205396 | 0 | 10 | 0.264842 | 0 | 4.534333 | 1.3E-07 | 0.046111 |
| 76 | 181 | 0.871391 | 2.128595 | 2.833333 | 22.16667 | 0.290465 | 3.251506 | 10.41376 | 2.06E-07 | 0.0363 |
| 77 | 185 | 0.606695 | 2.362158 | 1.333333 | 22.66667 | 0.204353 | 2.197698 | 9.595744 | 2.12E-07 | 0.054821 |
| 78 | 188 | 0.764318 | 2.217508 | 4 | 27 | 0.256325 | 5.233425 | 12.17583 | 1.99E-07 | 0.071598 |
| 79 | 190 | 0.843421 | 2.156498 | 6.333333 | 30.66667 | 0.281148 | 7.509099 | 14.22059 | 1.93E-07 | 0.095475 |
| 80 | 192 | 0.839121 | 2.124396 | 5.083333 | 30.91667 | 0.28315 | 6.057929 | 14.55316 | 2.44E-07 | 0.038887 |
| 81 | 197 | 0.897299 | 2.092858 | 4 | 21 | 0.300084 | 4.457824 | 10.03413 | 1.6E-07 | 0.090327 |
| 82 | 199 | 0.739864 | 2.247733 | 3.333333 | 26.66667 | 0.247645 | 4.505333 | 11.86381 | 2.11E-07 | 0.060826 |
| 83 | 201 | 0.886708 | 2.113292 | 2 | 21 | 0.295569 | 2.255535 | 9.937101 | 2.21E-07 | 0.017464 |
| 84 | 203 | 0.871251 | 2.128658 | 1.166667 | 33.83333 | 0.290426 | 1.339071 | 15.8942 | 4.18E-07 | 0.000195 |
| 85 | 204 | 0.89712 | 2.102865 | 2 | 16 | 0.299041 | 2.229357 | 7.608666 | 1.54E-07 | 0.060962 |
| 86 | 209 | 0.839349 | 2.12422 | 5.5 | 31.5 | 0.283222 | 6.552695 | 14.82897 | 2.38E-07 | 0.048296 |
| 87 | 210 | 0.663961 | 2.280787 | 3.083333 | 36.91667 | 0.225473 | 4.643845 | 16.18593 | 3.31E-07 | 0.013741 |
| 88 | 215 | 0.764654 | 2.21552 | 2.083333 | 18.91667 | 0.25658 | 2.724542 | 8.538251 | 1.67E-07 | 0.074753 |
| 89 | 217 | 0.886604 | 2.11335 | 1.666667 | 27.33333 | 0.295539 | 1.879832 | 12.93365 | 3.17E-07 | 0.002354 |
| 90 | 220 | 0.890027 | 2.049628 | 4.75 | 25.25 | 0.302766 | 5.336917 | 12.31931 | 2E-07 | 0.061108 |
| 91 | 221 | 0.894078 | 2.105739 | 4.583333 | 31.41667 | 0.298044 | 5.126323 | 14.91954 | 2.81E-07 | 0.016493 |
| 92 | 223 | 0.759599 | 2.222981 | 2 | 36 | 0.254678 | 2.632968 | 16.19447 | 3.89E-07 | 0.001354 |
| 93 | 224 | 0.788602 | 2.140093 | 5.083333 | 35.91667 | 0.269267 | 6.446006 | 16.78276 | 2.97E-07 | 0.022205 |
| 94 | 230 | 0.841059 | 2.096604 | 5 | 26 | 0.286302 | 5.944884 | 12.40101 | 1.85E-07 | 0.085114 |
| 95 | 234 | 0.843584 | 2.156387 | 2.5 | 19.5 | 0.281197 | 2.963545 | 9.042905 | 1.75E-07 | 0.063787 |
| 96 | 240 | 0.665837 | 2.271831 | 3.75 | 30.25 | 0.226655 | 5.63201 | 13.31525 | 2.21E-07 | 0.074729 |
| 97 | 242 | 0.858949 | 2.136014 | 6.333333 | 29.66667 | 0.286798 | 7.373351 | 13.8888 | 1.87E-07 | 0.098999 |
| 98 | 248 | 0.740316 | 2.247337 | 3.833333 | 29.16667 | 0.247792 | 5.177967 | 12.97832 | 2.24E-07 | 0.055268 |
| 99 | 249 | 0.39811 | 2.542659 | 0 | 20 | 0.135376 | 0 | 7.865781 | 2.26E-07 | 0.054519 |
| 100 | 251 | 0.788589 | 2.14006 | 4.583333 | 35.41667 | 0.269267 | 5.81207 | 16.54938 | 3.08E-07 | 0.017431 |
| 101 | 254 | 0.606618 | 2.362135 | 1 | 18 | 0.204334 | 1.648483 | 7.620224 | 1.71E-07 | 0.076421 |
| 102 | 257 | 0.755816 | 2.207702 | 4 | 28 | 0.25504 | 5.292297 | 12.68287 | 2.12E-07 | 0.061779 |
| 103 | 258 | 0.793641 | 2.180397 | 4.583333 | 30.41667 | 0.266856 | 5.775073 | 13.95006 | 2.35E-07 | 0.048525 |
| 104 | 263 | 0.745942 | 2.213701 | 3.833333 | 29.16667 | 0.252038 | 5.138918 | 13.17552 | 2.31E-07 | 0.049618 |
| 105 | 265 | 0.842629 | 2.031508 | 3.333333 | 28.66667 | 0.293176 | 3.955873 | 14.11103 | 2.92E-07 | 0.012177 |
| 106 | 266 | 0.886479 | 2.113444 | 3.166667 | 32.83333 | 0.295501 | 3.572183 | 15.53546 | 3.43E-07 | 0.003198 |
| 107 | 268 | 0.397888 | 2.552383 | 0.333333 | 29.66667 | 0.134865 | 0.837757 | 11.62312 | 3.1E-07 | 0.033157 |
| 108 | 270 | 0.865026 | 2.134944 | 2 | 23 | 0.288345 | 2.31207 | 10.77311 | 2.43E-07 | 0.012236 |
| 109 | 274 | 0.482966 | 2.457845 | 1 | 22 | 0.164229 | 2.07054 | 8.950931 | 1.98E-07 | 0.089106 |
| 110 | 276 | 0.88643 | 2.113545 | 2.5 | 23.5 | 0.295479 | 2.820302 | 11.11876 | 2.38E-07 | 0.018309 |
| 111 | 279 | 0.818304 | 2.147596 | 2.75 | 27.25 | 0.275904 | 3.360608 | 12.68861 | 2.68E-07 | 0.015182 |
| 112 | 280 | 0.82147 | 2.178484 | 5 | 27 | 0.273828 | 6.086651 | 12.39394 | 1.81E-07 | 0.093375 |
| 113 | 281 | 0.663912 | 2.280851 | 2.5 | 37.5 | 0.225455 | 3.765561 | 16.44123 | 3.64E-07 | 0.007313 |
| 114 | 282 | 0.859575 | 2.140362 | 1 | 25 | 0.286531 | 1.163366 | 11.68027 | 3.02E-07 | 0.001763 |
| 115 | 285 | 0.658872 | 2.312727 | 2.333333 | 25.66667 | 0.221723 | 3.541405 | 11.09801 | 2.17E-07 | 0.058082 |
| 116 | 286 | 0.839428 | 2.124138 | 3.25 | 30.75 | 0.283249 | 3.871686 | 14.47646 | 3.04E-07 | 0.009119 |
| 117 | 288 | 0.738163 | 2.244392 | 3.666667 | 34.33333 | 0.247494 | 4.967283 | 15.29739 | 2.97E-07 | 0.019594 |
| 118 | 290 | 0.738212 | 2.2351 | 3.666667 | 31.33333 | 0.248279 | 4.966954 | 14.01876 | 2.6E-07 | 0.033458 |
| 119 | 292 | 0.863027 | 2.136848 | 2.916667 | 31.08333 | 0.287688 | 3.379579 | 14.54635 | 3.21E-07 | 0.004578 |
| 120 | 293 | 0.896953 | 2.093104 | 2.5 | 22.5 | 0.299979 | 2.787214 | 10.74958 | 2.29E-07 | 0.021111 |
| 121 | 295 | 0.821365 | 2.105895 | 0.75 | 25.25 | 0.280592 | 0.913115 | 11.99015 | 3.18E-07 | 0.001645 |
| 122 | 296 | 0.756074 | 2.207496 | 4.25 | 31.75 | 0.255123 | 5.621143 | 14.38281 | 2.52E-07 | 0.040453 |
| 123 | 303 | 0.886554 | 2.113378 | 3.333333 | 29.66667 | 0.295525 | 3.759876 | 14.03756 | 2.95E-07 | 0.008901 |
| 124 | 311 | 0.780146 | 2.183428 | 2.333333 | 35.66667 | 0.263245 | 2.990894 | 16.33517 | 3.83E-07 | 0.002113 |
| 125 | 317 | 0.795027 | 2.102673 | 3 | 22 | 0.274365 | 3.773456 | 10.46287 | 1.92E-07 | 0.058555 |
| 126 | 318 | 0.871137 | 2.128806 | 3.166667 | 30.83333 | 0.290385 | 3.635095 | 14.48386 | 3.11E-07 | 0.006331 |
| 127 | 319 | 0.818522 | 2.14734 | 0 | 28 | 0.275981 | 0 | 13.03939 | 3.74E-07 | 0.000118 |
| 128 | 324 | 0.874311 | 2.115778 | 2 | 27 | 0.292403 | 2.287517 | 12.76127 | 3.01E-07 | 0.003625 |
| 129 | 327 | 0.475176 | 2.466823 | 1 | 22 | 0.161515 | 2.104481 | 8.918354 | 1.96E-07 | 0.094456 |
| 130 | 328 | 0.79226 | 2.193655 | 3.75 | 31.25 | 0.265332 | 4.733296 | 14.24563 | 2.73E-07 | 0.02196 |
| 131 | 330 | 0.85699 | 2.13247 | 2.666667 | 20.33333 | 0.28667 | 3.111667 | 9.535109 | 1.84E-07 | 0.053949 |
| 132 | 332 | 0.865056 | 2.134896 | 2.833333 | 21.16667 | 0.288357 | 3.275319 | 9.914611 | 1.91E-07 | 0.047891 |
| 133 | 335 | 0.886574 | 2.113399 | 3.166667 | 25.83333 | 0.295527 | 3.571803 | 12.22359 | 2.48E-07 | 0.018473 |
| 134 | 342 | 0.676467 | 2.306116 | 3.833333 | 31.16667 | 0.226806 | 5.666699 | 13.51479 | 2.25E-07 | 0.067769 |
| 135 | 343 | 0.818273 | 2.147579 | 4 | 23 | 0.275898 | 4.888344 | 10.70973 | 1.67E-07 | 0.097348 |
| 136 | 344 | 0.663939 | 2.28079 | 2.916667 | 37.08333 | 0.225467 | 4.392975 | 16.25898 | 3.41E-07 | 0.011011 |
| 137 | 346 | 0.897139 | 2.102771 | 3.5 | 29.5 | 0.299055 | 3.901289 | 14.02911 | 2.91E-07 | 0.009803 |
| 138 | 348 | 0.794935 | 2.205023 | 0.5 | 11.5 | 0.264982 | 0.628983 | 5.215364 | 1.32E-07 | 0.078647 |
| 139 | 349 | 0.818539 | 2.147377 | 2.333333 | 27.66667 | 0.275982 | 2.850609 | 12.88394 | 2.88E-07 | 0.009339 |
| 140 | 353 | 0.818019 | 2.147852 | 0 | 29 | 0.275811 | 0 | 13.50186 | 3.88E-07 | 8.62E-05 |
| 141 | 357 | 0.398027 | 2.552267 | 0.5 | 28.5 | 0.134911 | 1.256196 | 11.16654 | 2.85E-07 | 0.04877 |
| 142 | 359 | 0.886404 | 2.113515 | 1.75 | 33.25 | 0.295476 | 1.97427 | 15.73209 | 3.95E-07 | 0.000447 |
| 143 | 361 | 0.752606 | 2.18076 | 5.75 | 32.25 | 0.256567 | 7.640119 | 14.78842 | 2.05E-07 | 0.095316 |
| 144 | 366 | 0.756265 | 2.207321 | 5.333333 | 33.66667 | 0.255186 | 7.052204 | 15.25228 | 2.35E-07 | 0.062194 |
| 145 | 370 | 0.88636 | 2.113458 | 3.333333 | 25.66667 | 0.295471 | 3.760699 | 12.14439 | 2.41E-07 | 0.023255 |
| 146 | 371 | 0.663931 | 2.280842 | 1.5 | 36.5 | 0.225461 | 2.25927 | 16.00286 | 3.95E-07 | 0.002543 |
| 147 | 376 | 0.794819 | 2.124103 | 3 | 26 | 0.272299 | 3.774445 | 12.24046 | 2.43E-07 | 0.025814 |
| 148 | 377 | 0.818252 | 2.147586 | 2.333333 | 22.66667 | 0.275892 | 2.851608 | 10.55449 | 2.21E-07 | 0.030166 |
| 149 | 378 | 0.801606 | 2.115504 | 2.583333 | 25.41667 | 0.274795 | 3.222695 | 12.01447 | 2.52E-07 | 0.021037 |
| 150 | 380 | 0.737879 | 2.212458 | 4.333333 | 27.66667 | 0.2501 | 5.872686 | 12.50495 | 1.9E-07 | 0.097295 |
| 151 | 381 | 0.801801 | 2.115571 | 2.083333 | 23.91667 | 0.274837 | 2.598316 | 11.30507 | 2.5E-07 | 0.016303 |
| 152 | 382 | 0.863283 | 2.136707 | 3 | 27 | 0.287762 | 3.475106 | 12.63626 | 2.63E-07 | 0.01334 |
| 153 | 383 | 0.897428 | 2.102559 | 3 | 26 | 0.299144 | 3.342886 | 12.36588 | 2.59E-07 | 0.012393 |
| 154 | 384 | 0.557595 | 2.40456 | 0 | 34 | 0.18824 | 0 | 14.1398 | 4.06E-07 | 0.000833 |
| 155 | 387 | 0.755977 | 2.2076 | 4.583333 | 36.41667 | 0.255089 | 6.062796 | 16.49604 | 3E-07 | 0.022767 |
| 156 | 390 | 0.7884 | 2.140209 | 5.75 | 36.25 | 0.269206 | 7.293252 | 16.9376 | 2.77E-07 | 0.034954 |
| 157 | 396 | 0.842535 | 2.031595 | 5.833333 | 27.16667 | 0.293144 | 6.923547 | 13.37208 | 1.85E-07 | 0.099304 |
| 158 | 397 | 0.663912 | 2.280827 | 4.333333 | 36.66667 | 0.225457 | 6.526971 | 16.07604 | 2.74E-07 | 0.045025 |
| 159 | 400 | 0.788614 | 2.140079 | 3.833333 | 37.16667 | 0.269272 | 4.860846 | 17.36696 | 3.59E-07 | 0.005751 |
| 160 | 403 | 0.88971 | 2.110283 | 0 | 26 | 0.296571 | 0 | 12.32062 | 3.54E-07 | 0.000107 |
| 161 | 404 | 0.897445 | 2.102473 | 5.583333 | 30.41667 | 0.299157 | 6.221367 | 14.46709 | 2.37E-07 | 0.041142 |
| 162 | 405 | 0.398245 | 2.552026 | 0.5 | 25.5 | 0.134986 | 1.255509 | 9.992062 | 2.51E-07 | 0.0698 |
| 163 | 406 | 0.764893 | 2.216009 | 3.333333 | 32.66667 | 0.256598 | 4.357907 | 14.74121 | 2.98E-07 | 0.01528 |
| 164 | 410 | 0.735443 | 2.247157 | 3 | 30 | 0.246578 | 4.079172 | 13.3502 | 2.66E-07 | 0.02273 |
| 165 | 413 | 0.788587 | 2.140102 | 5 | 35 | 0.269263 | 6.340455 | 16.35436 | 2.88E-07 | 0.024049 |
| 166 | 416 | 0.865265 | 2.134705 | 1.583333 | 23.41667 | 0.288424 | 1.829883 | 10.96951 | 2.62E-07 | 0.00806 |
| 167 | 418 | 0.873049 | 2.116415 | 6.25 | 31.75 | 0.292042 | 7.158819 | 15.00179 | 2.25E-07 | 0.057657 |
| 168 | 423 | 0.756749 | 2.19769 | 2.5 | 29.5 | 0.25614 | 3.303606 | 13.42318 | 2.91E-07 | 0.013288 |
| 169 | 424 | 0.827232 | 2.158434 | 2 | 27 | 0.277068 | 2.4177 | 12.50907 | 2.9E-07 | 0.005884 |
| 170 | 425 | 0.843577 | 2.15632 | 2.75 | 21.25 | 0.281202 | 3.259927 | 9.854752 | 1.89E-07 | 0.051936 |
| 171 | 428 | 0.837283 | 2.110238 | 3.833333 | 26.16667 | 0.284063 | 4.578303 | 12.39986 | 2.25E-07 | 0.039898 |
| 172 | 429 | 0.897054 | 2.068787 | 3.5 | 23.5 | 0.302462 | 3.901662 | 11.35931 | 2.14E-07 | 0.037486 |
| 173 | 430 | 0.873371 | 1.936385 | 4.25 | 23.75 | 0.310835 | 4.866204 | 12.26512 | 2.13E-07 | 0.050436 |
| 174 | 433 | 0.557512 | 2.404666 | 0 | 42 | 0.18821 | 0 | 17.46605 | 5.01E-07 | 0.000157 |
| 175 | 434 | 0.897468 | 2.102522 | 1.5 | 25.5 | 0.299157 | 1.671369 | 12.12829 | 3E-07 | 0.003021 |
| 176 | 435 | 0.76123 | 2.221358 | 3.5 | 29.5 | 0.255225 | 4.597823 | 13.28017 | 2.49E-07 | 0.034458 |
| 177 | 440 | 0.788652 | 2.140023 | 2.916667 | 37.08333 | 0.269286 | 3.698295 | 17.32848 | 3.91E-07 | 0.00204 |
| 178 | 446 | 0.39813 | 2.475969 | 0.333333 | 24.66667 | 0.138523 | 0.837247 | 9.962431 | 2.62E-07 | 0.056273 |
| 179 | 454 | 0.865081 | 2.134847 | 0.333333 | 23.66667 | 0.288367 | 0.38532 | 11.08588 | 3.07E-07 | 0.001207 |
| 180 | 455 | 0.897465 | 2.10253 | 3 | 29 | 0.299156 | 3.342748 | 13.79291 | 3E-07 | 0.005634 |
| 181 | 458 | 0.663924 | 2.280794 | 1.333333 | 39.66667 | 0.225463 | 2.008263 | 17.39161 | 4.42E-07 | 0.001019 |
| 182 | 461 | 0.663874 | 2.280847 | 1.666667 | 40.33333 | 0.225445 | 2.510517 | 17.68349 | 4.36E-07 | 0.001354 |
| 183 | 462 | 0.795122 | 2.204878 | 0 | 14 | 0.265041 | 0 | 6.349559 | 1.82E-07 | 0.013418 |
| 184 | 468 | 0.886726 | 2.113249 | 4.333333 | 29.66667 | 0.295578 | 4.886891 | 14.03841 | 2.63E-07 | 0.021454 |
| 185 | 470 | 0.655185 | 2.318115 | 3 | 28 | 0.220356 | 4.578861 | 12.07878 | 2.15E-07 | 0.066101 |
| 186 | 471 | 0.81842 | 2.147351 | 1 | 26 | 0.275955 | 1.221866 | 12.10794 | 3.13E-07 | 0.001847 |
| 187 | 472 | 0.744136 | 2.215472 | 3.833333 | 29.16667 | 0.251431 | 5.151385 | 13.16499 | 2.3E-07 | 0.050395 |
| 188 | 474 | 0.896851 | 2.068972 | 0 | 18 | 0.302395 | 0 | 8.699974 | 2.5E-07 | 0.001531 |
| 189 | 478 | 0.788626 | 2.140043 | 4.5 | 37.5 | 0.269278 | 5.706127 | 17.52301 | 3.39E-07 | 0.01073 |
| 190 | 481 | 0.886605 | 2.113362 | 3 | 29 | 0.295538 | 3.383694 | 13.72221 | 2.97E-07 | 0.006333 |
| 191 | 487 | 0.691091 | 2.249716 | 1.666667 | 25.33333 | 0.235 | 2.411645 | 11.26068 | 2.54E-07 | 0.02267 |
| 192 | 488 | 0.78854 | 2.14014 | 4.166667 | 35.83333 | 0.269248 | 5.284025 | 16.74345 | 3.29E-07 | 0.010824 |
| 193 | 491 | 0.756142 | 2.207411 | 5.083333 | 34.91667 | 0.255147 | 6.722721 | 15.81793 | 2.61E-07 | 0.041091 |
| 194 | 493 | 0.803108 | 2.184509 | 3.833333 | 27.16667 | 0.268812 | 4.77312 | 12.43605 | 2.2E-07 | 0.047385 |
| 195 | 494 | 0.794837 | 2.205115 | 2.333333 | 18.66667 | 0.26495 | 2.935612 | 8.465166 | 1.59E-07 | 0.088225 |
| 196 | 497 | 0.815339 | 2.14825 | 3 | 30 | 0.275119 | 3.67945 | 13.96486 | 2.95E-07 | 0.009495 |
| 197 | 500 | 0.788743 | 2.139947 | 3.75 | 36.25 | 0.269316 | 4.754402 | 16.93967 | 3.5E-07 | 0.006663 |
| 198 | 501 | 0.794573 | 2.205252 | 2.5 | 19.5 | 0.264873 | 3.146346 | 8.842527 | 1.64E-07 | 0.08603 |
| 199 | 502 | 0.793729 | 2.180354 | 3.333333 | 28.66667 | 0.266882 | 4.199584 | 13.14771 | 2.57E-07 | 0.025606 |
| 200 | 503 | 0.838007 | 2.137656 | 2.416667 | 35.58333 | 0.28162 | 2.883827 | 16.64596 | 3.95E-07 | 0.001168 |
| 201 | 506 | 0.842542 | 2.031618 | 3.333333 | 22.66667 | 0.293144 | 3.956279 | 11.15695 | 2.07E-07 | 0.048445 |
| 202 | 518 | 0.663872 | 2.280843 | 0.75 | 39.25 | 0.225445 | 1.129736 | 17.20855 | 4.62E-07 | 0.000355 |
| 203 | 524 | 0.788459 | 2.140217 | 3.666667 | 37.33333 | 0.26922 | 4.650419 | 17.44372 | 3.67E-07 | 0.004953 |
| 204 | 527 | 0.788677 | 2.169413 | 3.083333 | 36.91667 | 0.266617 | 3.909499 | 17.01689 | 3.76E-07 | 0.002981 |
| 205 | 528 | 0.794812 | 2.20514 | 1 | 15 | 0.264942 | 1.258159 | 6.80229 | 1.59E-07 | 0.049152 |
| 206 | 529 | 0.738237 | 2.212112 | 5.166667 | 34.83333 | 0.25022 | 6.99866 | 15.74664 | 2.51E-07 | 0.051779 |
| 207 | 530 | 0.691614 | 2.260607 | 0.666667 | 27.33333 | 0.234269 | 0.963929 | 12.09115 | 3.19E-07 | 0.00381 |
| 208 | 538 | 0.79493 | 2.124043 | 4 | 28 | 0.272332 | 5.031888 | 13.18241 | 2.34E-07 | 0.040012 |
| 209 | 540 | 0.818526 | 2.14737 | 2 | 28 | 0.275979 | 2.443416 | 13.03921 | 3.04E-07 | 0.004691 |
| 210 | 541 | 0.676543 | 2.306025 | 4.416667 | 32.58333 | 0.226832 | 6.528289 | 14.12965 | 2.18E-07 | 0.084452 |
| 211 | 550 | 0.839373 | 2.12425 | 5 | 27 | 0.283225 | 5.95683 | 12.71037 | 1.94E-07 | 0.075723 |
| 212 | 552 | 0.886718 | 2.113258 | 2.833333 | 30.16667 | 0.295575 | 3.195304 | 14.27496 | 3.18E-07 | 0.004223 |
| 213 | 553 | 0.880245 | 2.119666 | 3.416667 | 30.58333 | 0.293424 | 3.881493 | 14.42837 | 3.03E-07 | 0.008293 |
| 214 | 554 | 0.86312 | 2.13687 | 3 | 26 | 0.287708 | 3.475763 | 12.16733 | 2.5E-07 | 0.017057 |
| 215 | 555 | 0.557577 | 2.404614 | 1 | 41 | 0.188231 | 1.793473 | 17.05056 | 4.38E-07 | 0.001687 |
| 216 | 556 | 0.796417 | 2.122893 | 6.25 | 31.75 | 0.27281 | 7.847645 | 14.956 | 2.04E-07 | 0.092595 |
| 217 | 560 | 0.886568 | 2.113353 | 3.833333 | 22.16667 | 0.29553 | 4.32379 | 10.48886 | 1.77E-07 | 0.071657 |
| 218 | 561 | 0.794811 | 2.124108 | 4 | 28 | 0.272296 | 5.032644 | 13.18201 | 2.34E-07 | 0.040049 |
| 219 | 563 | 0.897142 | 2.06869 | 5 | 26 | 0.302493 | 5.573253 | 12.56834 | 2.01E-07 | 0.059159 |
| 220 | 564 | 0.780566 | 2.19353 | 3.5 | 29.5 | 0.262455 | 4.483923 | 13.44864 | 2.57E-07 | 0.028333 |
| 221 | 565 | 0.764707 | 2.215406 | 2.5 | 25.5 | 0.256603 | 3.269226 | 11.5103 | 2.37E-07 | 0.030502 |
| 222 | 566 | 0.897184 | 2.068568 | 4.75 | 28.25 | 0.302515 | 5.294345 | 13.65679 | 2.4E-07 | 0.03254 |
| 223 | 568 | 0.691553 | 2.249284 | 1.166667 | 29.83333 | 0.235155 | 1.687023 | 13.26348 | 3.32E-07 | 0.004391 |
| 224 | 571 | 0.398314 | 2.475758 | 1.166667 | 27.83333 | 0.138589 | 2.929015 | 11.24235 | 2.39E-07 | 0.098027 |
| 225 | 572 | 0.865094 | 2.134876 | 3.333333 | 20.66667 | 0.288368 | 3.853147 | 9.680501 | 1.67E-07 | 0.08144 |
| 226 | 577 | 0.886689 | 2.113289 | 3 | 30 | 0.295565 | 3.383373 | 14.19588 | 3.1E-07 | 0.004864 |
| 227 | 578 | 0.897363 | 2.102575 | 2.833333 | 28.16667 | 0.299127 | 3.1574 | 13.39627 | 2.94E-07 | 0.006398 |
| 228 | 580 | 0.788625 | 2.140075 | 3.333333 | 34.66667 | 0.269275 | 4.226765 | 16.19881 | 3.44E-07 | 0.006557 |
| 229 | 581 | 0.421195 | 2.559017 | 0.833333 | 27.16667 | 0.14133 | 1.978499 | 10.61605 | 2.48E-07 | 0.067923 |
| 230 | 586 | 0.752668 | 2.180592 | 2.75 | 38.25 | 0.256598 | 3.653671 | 17.54111 | 3.99E-07 | 0.002319 |
| 231 | 587 | 0.880421 | 2.119547 | 3.833333 | 25.16667 | 0.293477 | 4.353979 | 11.8736 | 2.16E-07 | 0.039366 |
| 232 | 588 | 0.676613 | 2.306035 | 3.833333 | 34.16667 | 0.22685 | 5.665471 | 14.8162 | 2.63E-07 | 0.042094 |
| 233 | 590 | 0.839274 | 2.124303 | 4.583333 | 33.41667 | 0.283196 | 5.46107 | 15.73065 | 2.95E-07 | 0.016843 |
| 234 | 595 | 0.663862 | 2.280894 | 2.333333 | 33.66667 | 0.225439 | 3.514786 | 14.7603 | 3.23E-07 | 0.012525 |
| 235 | 597 | 0.699945 | 2.240196 | 4.25 | 34.75 | 0.238065 | 6.071904 | 15.51204 | 2.71E-07 | 0.039135 |
| 236 | 601 | 0.831922 | 2.143781 | 2.5 | 21.5 | 0.279572 | 3.005088 | 10.02901 | 2.02E-07 | 0.04242 |
| 237 | 602 | 0.865028 | 2.134811 | 2.833333 | 23.16667 | 0.288358 | 3.275424 | 10.85186 | 2.18E-07 | 0.030132 |
| 238 | 603 | 0.66346 | 2.281324 | 2.833333 | 36.16667 | 0.2253 | 4.270544 | 15.85337 | 3.33E-07 | 0.012447 |
| 239 | 604 | 0.757338 | 2.215963 | 5.083333 | 32.91667 | 0.254713 | 6.712106 | 14.85434 | 2.34E-07 | 0.058348 |
| 240 | 605 | 0.859088 | 2.140878 | 3 | 28 | 0.286366 | 3.492074 | 13.07874 | 2.75E-07 | 0.010867 |
| 241 | 609 | 0.818455 | 2.147409 | 3 | 28 | 0.275959 | 3.665441 | 13.03897 | 2.69E-07 | 0.014797 |
| 242 | 611 | 0.871271 | 2.128694 | 5.083333 | 28.91667 | 0.290427 | 5.834391 | 13.58423 | 2.23E-07 | 0.047612 |
| 243 | 613 | 0.557512 | 2.404666 | 0 | 42 | 0.18821 | 0 | 17.46605 | 5.01E-07 | 0.000157 |
| 244 | 617 | 0.421279 | 2.558992 | 1 | 28 | 0.141356 | 2.373723 | 10.94181 | 2.46E-07 | 0.069514 |
| 245 | 619 | 0.726143 | 2.205243 | 2 | 35 | 0.247713 | 2.754278 | 15.87126 | 3.77E-07 | 0.002278 |
| 246 | 621 | 0.55786 | 2.404361 | 0 | 38 | 0.188325 | 0 | 15.80462 | 4.54E-07 | 0.00036 |
| 247 | 622 | 0.756136 | 2.207442 | 4.166667 | 35.83333 | 0.255143 | 5.510473 | 16.23297 | 3.08E-07 | 0.017402 |
| 248 | 623 | 0.39769 | 2.543026 | 0 | 17 | 0.135236 | 0 | 6.68495 | 1.92E-07 | 0.084579 |
| 249 | 626 | 0.691312 | 2.260626 | 2.666667 | 25.33333 | 0.234189 | 3.857398 | 11.20633 | 2.11E-07 | 0.061134 |
| 250 | 627 | 0.691219 | 2.249629 | 1 | 19 | 0.235041 | 1.44672 | 8.445838 | 2.01E-07 | 0.033634 |
| 251 | 628 | 0.803376 | 2.170656 | 4 | 29 | 0.27013 | 4.978986 | 13.36002 | 2.41E-07 | 0.034686 |
| 252 | 629 | 0.699843 | 2.240281 | 2.333333 | 34.66667 | 0.238032 | 3.334079 | 15.47425 | 3.49E-07 | 0.006702 |
| 253 | 634 | 0.691246 | 2.249554 | 0 | 26 | 0.235054 | 0 | 11.55785 | 3.32E-07 | 0.000943 |
| 254 | 635 | 0.397953 | 2.552348 | 0 | 27 | 0.134885 | 0 | 10.5785 | 3.04E-07 | 0.019998 |
| 255 | 637 | 0.795137 | 2.124045 | 4.5 | 28.5 | 0.272384 | 5.6594 | 13.4178 | 2.23E-07 | 0.056814 |
| 256 | 638 | 0.794975 | 2.102518 | 2 | 16 | 0.274367 | 2.515802 | 7.609922 | 1.46E-07 | 0.092335 |
| 257 | 640 | 0.765257 | 2.217409 | 4.833333 | 32.16667 | 0.256568 | 6.315959 | 14.50642 | 2.35E-07 | 0.053845 |
| 258 | 641 | 0.795148 | 2.102591 | 2 | 25 | 0.274403 | 2.515254 | 11.89009 | 2.69E-07 | 0.010642 |
| 259 | 647 | 0.691647 | 2.249092 | 2.75 | 28.25 | 0.235195 | 3.976018 | 12.56062 | 2.46E-07 | 0.037454 |
| 260 | 649 | 0.880324 | 2.021043 | 3 | 29 | 0.303417 | 3.407837 | 14.34903 | 3.14E-07 | 0.004901 |
| 261 | 651 | 0.557512 | 2.404666 | 0 | 42 | 0.18821 | 0 | 17.46605 | 5.01E-07 | 0.000157 |
| 262 | 652 | 0.700586 | 2.282066 | 3.833333 | 32.16667 | 0.234887 | 5.47161 | 14.09542 | 2.48E-07 | 0.046541 |
| 263 | 653 | 0.398021 | 2.552252 | 0 | 28 | 0.13491 | 0 | 10.9707 | 3.15E-07 | 0.017287 |
| 264 | 655 | 0.421241 | 2.558987 | 0 | 28 | 0.141345 | 0 | 10.94183 | 3.14E-07 | 0.014026 |
| 265 | 656 | 0.421624 | 2.558632 | 0 | 29 | 0.141472 | 0 | 11.33418 | 3.25E-07 | 0.011991 |
| 266 | 657 | 0.664065 | 2.280746 | 3.166667 | 33.83333 | 0.225504 | 4.768607 | 14.83433 | 2.89E-07 | 0.026551 |
| 267 | 658 | 0.76483 | 2.198531 | 4.5 | 29.5 | 0.258095 | 5.883662 | 13.41805 | 2.16E-07 | 0.067229 |
| 268 | 659 | 0.752198 | 2.181105 | 2.916667 | 29.08333 | 0.256434 | 3.877526 | 13.33422 | 2.72E-07 | 0.019635 |
| 269 | 660 | 0.690567 | 2.259609 | 3.416667 | 36.58333 | 0.234077 | 4.947624 | 16.19012 | 3.23E-07 | 0.016319 |
| 270 | 661 | 0.39769 | 2.552585 | 0 | 28 | 0.134798 | 0 | 10.96927 | 3.15E-07 | 0.01735 |
| 271 | 663 | 0.863338 | 2.136551 | 4.583333 | 26.41667 | 0.28779 | 5.30885 | 12.36416 | 2.03E-07 | 0.061829 |
| 272 | 665 | 0.897366 | 2.102616 | 7 | 30 | 0.299124 | 7.80061 | 14.26794 | 1.86E-07 | 0.096638 |
| 273 | 671 | 0.818457 | 2.147455 | 2 | 27 | 0.275955 | 2.443623 | 12.57302 | 2.91E-07 | 0.00609 |
| 274 | 673 | 0.397717 | 2.543075 | 0 | 16 | 0.135241 | 0 | 6.291597 | 1.81E-07 | 0.097795 |
| 275 | 674 | 0.842757 | 2.031382 | 6.666667 | 30.33333 | 0.293221 | 7.910546 | 14.93236 | 2.02E-07 | 0.091139 |
| 276 | 676 | 0.81828 | 2.147537 | 3 | 28 | 0.275904 | 3.666225 | 13.03819 | 2.69E-07 | 0.014821 |
| 277 | 678 | 0.897304 | 2.102636 | 4.5 | 30.5 | 0.299107 | 5.01502 | 14.5056 | 2.72E-07 | 0.018486 |
| 278 | 681 | 0.764581 | 2.215638 | 1.5 | 38.5 | 0.256552 | 1.961859 | 17.37649 | 4.43E-07 | 0.000434 |
| 279 | 687 | 0.865272 | 2.134724 | 0.5 | 15.5 | 0.288424 | 0.577853 | 7.260892 | 1.92E-07 | 0.018331 |
| 280 | 690 | 0.421727 | 2.558563 | 0 | 25 | 0.141505 | 0 | 9.77111 | 2.81E-07 | 0.022052 |
| 281 | 692 | 0.802416 | 2.183462 | 1.5 | 15.5 | 0.268737 | 1.869355 | 7.098817 | 1.5E-07 | 0.080338 |
| 282 | 698 | 0.756619 | 2.197813 | 3 | 29 | 0.256096 | 3.965005 | 13.19494 | 2.65E-07 | 0.021133 |
| 283 | 700 | 0.663881 | 2.280871 | 3.5 | 38.5 | 0.225446 | 5.272026 | 16.87952 | 3.33E-07 | 0.016873 |
| 284 | 701 | 0.897421 | 2.068296 | 6.75 | 29.25 | 0.302598 | 7.521552 | 14.14208 | 1.9E-07 | 0.092146 |
| 285 | 704 | 0.818279 | 2.147538 | 2 | 28 | 0.275903 | 2.444153 | 13.03819 | 3.04E-07 | 0.004703 |
| 286 | 708 | 0.39769 | 2.552585 | 0 | 28 | 0.134798 | 0 | 10.96927 | 3.15E-07 | 0.01735 |
| 287 | 709 | 0.557512 | 2.404666 | 0 | 42 | 0.18821 | 0 | 17.46605 | 5.01E-07 | 0.000157 |
| 288 | 713 | 0.397717 | 2.552557 | 0 | 28 | 0.134807 | 0 | 10.96939 | 3.15E-07 | 0.017345 |
| 289 | 714 | 0.729571 | 2.228569 | 5 | 37 | 0.246632 | 6.853344 | 16.60258 | 2.8E-07 | 0.034101 |
| 290 | 719 | 0.817905 | 2.147827 | 3 | 28 | 0.275785 | 3.667909 | 13.03643 | 2.69E-07 | 0.014872 |
| 291 | 720 | 0.863219 | 2.136684 | 4 | 29 | 0.287749 | 4.633817 | 13.57243 | 2.57E-07 | 0.021248 |
| 292 | 721 | 0.886679 | 2.113321 | 4 | 27 | 0.29556 | 4.511217 | 12.7761 | 2.37E-07 | 0.026928 |
| 293 | 722 | 0.81834 | 2.14756 | 2.25 | 26.75 | 0.275916 | 2.749467 | 12.456 | 2.79E-07 | 0.010438 |
| 294 | 731 | 0.663808 | 2.280918 | 2.083333 | 39.91667 | 0.225423 | 3.13846 | 17.50026 | 4.12E-07 | 0.002405 |
| 295 | 732 | 0.886456 | 2.113515 | 1.5 | 25.5 | 0.295488 | 1.692132 | 12.06521 | 2.98E-07 | 0.003376 |
| 296 | 734 | 0.755712 | 2.207811 | 2.25 | 36.75 | 0.255005 | 2.977323 | 16.64545 | 3.92E-07 | 0.001991 |
| 297 | 735 | 0.397698 | 2.552577 | 0 | 28 | 0.1348 | 0 | 10.96931 | 3.15E-07 | 0.017348 |
| 298 | 739 | 0.691234 | 2.288971 | 1.666667 | 19.33333 | 0.231942 | 2.411146 | 8.446298 | 1.73E-07 | 0.078825 |
| 299 | 740 | 0.872659 | 1.956062 | 4.5 | 27.5 | 0.3085 | 5.156653 | 14.05886 | 2.56E-07 | 0.028078 |
| 300 | 745 | 0.691354 | 2.249463 | 2.333333 | 27.66667 | 0.235089 | 3.375022 | 12.29923 | 2.56E-07 | 0.029204 |
| 301 | 749 | 0.756168 | 2.207388 | 5 | 37 | 0.255156 | 6.61229 | 16.76189 | 2.91E-07 | 0.025964 |
| 302 | 750 | 0.744381 | 2.255551 | 3.666667 | 28.33333 | 0.248133 | 4.925796 | 12.5616 | 2.19E-07 | 0.057459 |
| 303 | 759 | 0.818439 | 2.181489 | 2.583333 | 30.41667 | 0.27282 | 3.156416 | 13.94308 | 3.1E-07 | 0.006951 |
| 304 | 760 | 0.397709 | 2.543094 | 0 | 25 | 0.135238 | 0 | 9.830545 | 2.82E-07 | 0.026449 |
| 305 | 761 | 0.557512 | 2.404666 | 0 | 42 | 0.18821 | 0 | 17.46605 | 5.01E-07 | 0.000157 |
| 306 | 762 | 0.765324 | 2.198292 | 3.5 | 31.5 | 0.25824 | 4.573226 | 14.32931 | 2.8E-07 | 0.021479 |
| 307 | 767 | 0.397932 | 2.552305 | 0.666667 | 26.33333 | 0.134881 | 1.675329 | 10.31747 | 2.48E-07 | 0.07613 |
| 308 | 768 | 0.397792 | 2.476146 | 0 | 28 | 0.138414 | 0 | 11.3079 | 3.25E-07 | 0.01543 |
| 309 | 772 | 0.557512 | 2.404666 | 0 | 42 | 0.18821 | 0 | 17.46605 | 5.01E-07 | 0.000157 |
| 310 | 773 | 0.874075 | 2.104637 | 3.75 | 23.25 | 0.293441 | 4.290249 | 11.04704 | 1.94E-07 | 0.056962 |
| 311 | 775 | 0.756026 | 2.207486 | 3.916667 | 38.08333 | 0.255112 | 5.180595 | 17.2519 | 3.47E-07 | 0.008443 |
| 312 | 776 | 0.482359 | 2.46978 | 0 | 17 | 0.163393 | 0 | 6.883204 | 1.98E-07 | 0.04818 |
| 313 | 778 | 0.874576 | 2.125416 | 4.5 | 28.5 | 0.291526 | 5.145352 | 13.40914 | 2.37E-07 | 0.035157 |
| 314 | 779 | 0.848912 | 2.140459 | 3.916667 | 37.08333 | 0.283977 | 4.61375 | 17.32494 | 3.65E-07 | 0.003503 |
| 315 | 781 | 0.765172 | 2.198351 | 4.5 | 28.5 | 0.258197 | 5.881028 | 12.96426 | 2.03E-07 | 0.079336 |
