# Supplementary material for: Positive Selection of TLR2 and MyD88 Genes Provides Insights Into the Molecular Basis of Immunological Adaptation in Amphibians
Source: Ecol Evol. 2024 Dec 16;14(12):e70723. doi: 10.1002/ece3.70723 (PMC11650749; doi:10.1002/ece3.70723)
Supplement: Supplementary file 11 — Table S5. Positive selection sites for the MyD88 gene based on FEL analysis. [file ECE3-14-e70723-s002.docx]

Table S5. Positive selection sites for the MyD88 gene based on FEL analysis.

| NO. | Site | α | β | α=β | p-value | Estimate of parameters(ω) |
| --- | --- | --- | --- | --- | --- | --- |
| 1 | 279 | 0.358 | 0.98 | 0.721 | 0.1393 | 2.74 |
