# Supplementary material for: Positive Selection of TLR2 and MyD88 Genes Provides Insights Into the Molecular Basis of Immunological Adaptation in Amphibians
Source: Ecol Evol. 2024 Dec 16;14(12):e70723. doi: 10.1002/ece3.70723 (PMC11650749; doi:10.1002/ece3.70723)
Supplement: Supplementary file 12 — Table S6. Positive selection sites for the MyD88 gene based on MEME analysis. [file ECE3-14-e70723-s009.docx]

Table S6. Positive selection sites for the MyD88 gene based on MEME analysis.

| NO. | Site | α | β- | p- | β+ | p+ | p-value |
| --- | --- | --- | --- | --- | --- | --- | --- |
| 1 | 5 | 1.78 | 1.78 | 0 | 275.53 | 1 | 0.01 |
| 2 | 9 | 6.86 | 2.62 | 0 | 8147.16 | 1 | 0.02 |
| 3 | 13 | 1.48 | 0 | 0 | 40.31 | 1 | 0.1 |
| 4 | 37 | 0 | 0 | 0 | 12.95 | 1 | 0.09 |
| 5 | 38 | 3.87 | 0 | 0 | 199.59 | 1 | 0.01 |
| 6 | 67 | 4.58 | 2.35 | 0 | 245.54 | 1 | 0.04 |
| 7 | 77 | 5.64 | 0 | 0 | 127.82 | 1 | 0.08 |
| 8 | 80 | 3.96 | 0 | 0 | 59.45 | 1 | 0.05 |
| 9 | 149 | 3.12 | 0 | 0 | 100000 | 1 | 0.07 |
| 10 | 165 | 1.91 | 0.7 | 0 | 44.76 | 1 | 0.1 |
| 11 | 176 | 2.03 | 1.49 | 0 | 49.44 | 1 | 0.07 |
