# Supplementary material for: Positive Selection of TLR2 and MyD88 Genes Provides Insights Into the Molecular Basis of Immunological Adaptation in Amphibians
Source: Ecol Evol. 2024 Dec 16;14(12):e70723. doi: 10.1002/ece3.70723 (PMC11650749; doi:10.1002/ece3.70723)
Supplement: Supplementary file 6 — Figure S6. Multiple sequence alignments of the representative amphibian MyD88. The positions of the death domain and TIR domain are indicated. Positively selected sites are shown in yellow colors. Information of the sequences used and the positively selected sites are shown in Tables 1 and 4. [file ECE3-14-e70723-s003.pdf]

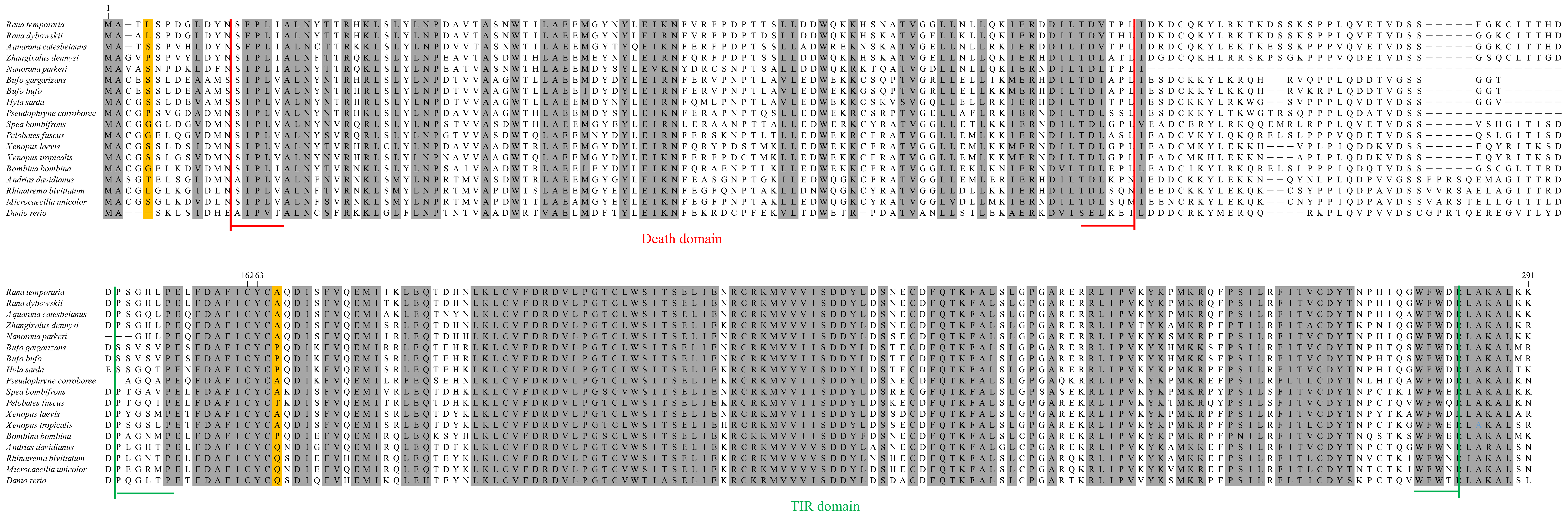
1
Rana temporaria
Rana dybowskii
Aquarana catesbeianus
Zhangixalus dennysi
Nanorana parkeri
Bufo gargarizans
Bufo bufo
Hyla sarda
Pseudophryne corroboree
Spea bombifrons
Pelobates fuscus
Xenopus laevis
Xenopus tropicalis
Bombina bombina
Andrias davidianus
Rhinatrema bivittatum
Microcaecilia unicolor
Danio rerio
Death domain
162 163
291
TIR domain
